# Olive phenolic compounds: metabolic and transcriptional profiling during fruit development

**DOI:** 10.1186/1471-2229-12-162

**Published:** 2012-09-10

**Authors:** Fiammetta Alagna, Roberto Mariotti, Francesco Panara, Silvia Caporali, Stefania Urbani, Gianluca Veneziani, Sonia Esposto, Agnese Taticchi, Adolfo Rosati, Rosa Rao, Gaetano Perrotta, Maurizio Servili, Luciana Baldoni

**Affiliations:** 1 CNR – Institute of Plant Genetics, 06128, Perugia, Italy; 2 Dept. of Economical and Food Science, University of Perugia, 06126, Perugia, Italy; 3 CRA –OLI, 06049, Spoleto, PG, Italy; 4 Dept. of Soil, Plant, Environment and Animal Production Sciences, University of Naples 'Federico II', 80055, Portici, NA, Italy; 5 ENEA, TRISAIA Research Center, 75026, Rotondella, Matera, Italy

**Keywords:** *Olea europaea*, Phenolics, Secoiridoids, RT-qPCR, Transcriptome, Secondary metabolism

## Abstract

**Background:**

Olive (*Olea europaea* L.) fruits contain numerous secondary metabolites, primarily phenolics, terpenes and sterols, some of which are particularly interesting for their nutraceutical properties. This study will attempt to provide further insight into the profile of olive phenolic compounds during fruit development and to identify the major genetic determinants of phenolic metabolism.

**Results:**

The concentration of the major phenolic compounds, such as oleuropein, demethyloleuropein, 3–4 DHPEA-EDA, ligstroside, tyrosol, hydroxytyrosol, verbascoside and lignans, were measured in the developing fruits of 12 olive cultivars. The content of these compounds varied significantly among the cultivars and decreased during fruit development and maturation, with some compounds showing specificity for certain cultivars. Thirty-five olive transcripts homologous to genes involved in the pathways of the main secondary metabolites were identified from the massive sequencing data of the olive fruit transcriptome or from cDNA-AFLP analysis. Their mRNA levels were determined using RT-qPCR analysis on fruits of high- and low-phenolic varieties (*Coratina* and *Dolce d’Andria*, respectively) during three different fruit developmental stages. A strong correlation was observed between phenolic compound concentrations and transcripts putatively involved in their biosynthesis, suggesting a transcriptional regulation of the corresponding pathways. *OeDXS*, *OeGES*, *OeGE10H* and *OeADH*, encoding putative 1-deoxy-D-xylulose-5-P synthase, geraniol synthase, geraniol 10-hydroxylase and arogenate dehydrogenase, respectively, were almost exclusively present at 45 days after flowering (DAF), suggesting that these compounds might play a key role in regulating secoiridoid accumulation during fruit development.

**Conclusions:**

Metabolic and transcriptional profiling led to the identification of some major players putatively involved in biosynthesis of secondary compounds in the olive tree. Our data represent the first step towards the functional characterisation of important genes for the determination of olive fruit quality.

## Background

The olive fruit mesocarp accumulates a wide range of secondary metabolites. The main category of secondary metabolites is represented by secoiridoids, a group of monoterpenoids with a cleaved methylcyclopentane skeleton, which are typical of the Oleaceae and other few dicotyledonous families. Secoiridoids are abundant in olives as phenol-conjugated compounds that might contain a glycoside moiety. The most important secoiridoids of the olive fruit and virgin olive oil are oleuropein, demethyloleuropein, oleuroside, ligstroside, nüzhenide and their aglycon forms, such as the dialdehydic form of decarboxymethyl elenolic acid linked to either 3,4-DHPEA or p-HPEA (3,4-DHPEA-EDA and p-HPEA-EDA, respectively), an isomer of oleuropein aglycon (3,4-DHPEA-EA) and the ligstroside aglycon (p-HPEA-EA) [1]. The secoiridoid compound p-HPEA-EDA, also called oleocanthal, was identified in the extra-virgin olive oil but has never been observed in the fruits, probably as a consequence of post-harvest enzymatic activity [2]. Other olive phenolics include phenolic acids, phenolic alcohols (e.g., hydroxytyrosol (3,4-DHPEA) and tyrosol (p-HPEA), flavonoids and lignans [3]. These compounds are observed in all parts of the drupe, with the highest concentration in the pulp [4]. The fruits of several olive cultivars also contain high amounts of verbascoside [5] and other phenolics present at maturity, such as homovanillic alcohol, 3,4-dihydroxyphenylacetic acid (DHPAC), caffeic acid, p-coumaric acid, phloretic acid, vanillic acid [6] and low amounts of comselogoside [7].

Studies of the phenolic profiles in the mesocarp, exocarp, seed, stone and leaf of the olive have demonstrated that different tissues contain distinct compounds. For example, nüzhenide and salidroside are only observed in the olive seed [8], while the flavonoids luteolin-7-glucoside, rutin and quercetin are exclusively present in the fruit peel [9].

Other compounds in the olive fruit include triterpenic acids such as maslinic and oleanolic acids [10] and tocopherols [11].

Squalene, an intermediate of the sterol pathway, is the precursor of α- and β-amyrins and the triterpenic diols erythrodiol and uvaol. Squalene is another important compound with recognised effects on human health that is present in consistent amount only in olive and other vegetable oils [12]. Sterols, such as β-sitosterol, cycloartenol and 24-methylenecycloartanol, progressively accumulate when the olive fruit reaches its final size and veraison begins [13].

Secoiridoids are not soluble in oil and, after the process of mechanical extraction, only a small portion is recovered in the oil, representing the most important microconstituents of virgin olive oils for their health and sensory proprieties [14]. Indeed, olive secoiridoids play a role in the prevention of atherosclerosis and the inhibition of low-density lipoprotein peroxidation [12]. Numerous studies have clearly demonstrated that these compounds exhibit cancer preventive activities [15] and can contribute to the nutritional prevention of osteoporosis [16]. In particular, oleuropein, hydroxytyrosol [15] and oleocanthal [2] have shown effects on human health. The secoiridoids contribute to the quality of olive oil, influencing the oil taste, being responsible for bitter and pungency sensory notes and as primary antioxidants, secoiridoids are involved in oil oxidative stability [5].

Phenolics play a crucial role in the plant response to environmental cues, being the most important defence compounds against defoliating insects [17]. They also affect shoot branching [18] and have been hypothesised to protect cells and prevent fungal penetration into the cambial zone [19,20]. Some data support the idea that the resistance to specific pathogens might also be related to certain types of phenolics [21,22]. Oleuropein is responsible for the release of phytoalexins [23], and it is also a multivalent alkylator that functions as an ideal protein cross-linker, exhibiting the strongest activity reported for a plant metabolite, which adversely affects herbivores by decreasing the nutritive value of dietary proteins [24].

To date, secoiridoid metabolism has not been well clarified, but a pathway has been proposed for some Oleaceae species [25,26]. Secoiridoid accumulation is a controlled process with expression and composition varying considerably among varieties, tissues, developmental stages and in response to different environmental conditions [27].

The key genes that modulate the synthesis and degradation of secondary compounds in olive fruits have not been characterised, with the exception of a few genes involved in triterpene biosynthesis [28,29], due to the lack of information for the olive genome sequence. The first olive fruit transcriptome data were recently released [30,31], representing an important resource for the identification of genes involved in fruit metabolism.

Functional genetic studies are difficult to perform in perennial woody species due to the lack of efficient protocols for mutagenesis, transformation and *in vitro* regeneration; therefore, understanding the natural variations for traits of interest represents a valuable tool. In plant science, the integration of gene expression and metabolic data sets is currently being attempted to study metabolic pathways [32].

The aim of this work was to provide further insight into the evolution of the olive fruit phenolic compounds and to identify their major genetic determinants.

## Results and discussion

### Concentration of phenolics according to variety and fruit developmental stage

The cultivars chosen for the analyses represent a high level of variation in the fruit phenolics content, based on a number of studies previously performed on subgroups of these compounds or empirical information, as is the case for *Dolce d’Andria* and *Tendellone*, which are traditionally used as table olives and are directly edible without undergoing the debittering process. To our knowledge, our work represents the first attempt to directly compare phenolic profiles of olive cultivars grown under the same environmental conditions. These cultivars, in fact, are cultivated in different regions of Italy, under different climate conditions, soils, water availabilities and agro-techniques that greatly affect the fruit phenolic content. A previous molecular characterisation of these varieties has established that they have originated independently, and no close genetic relationships have been observed among either the high- or low-phenolics cultivars (Baldoni, unpub. data).

**Figure 1:**
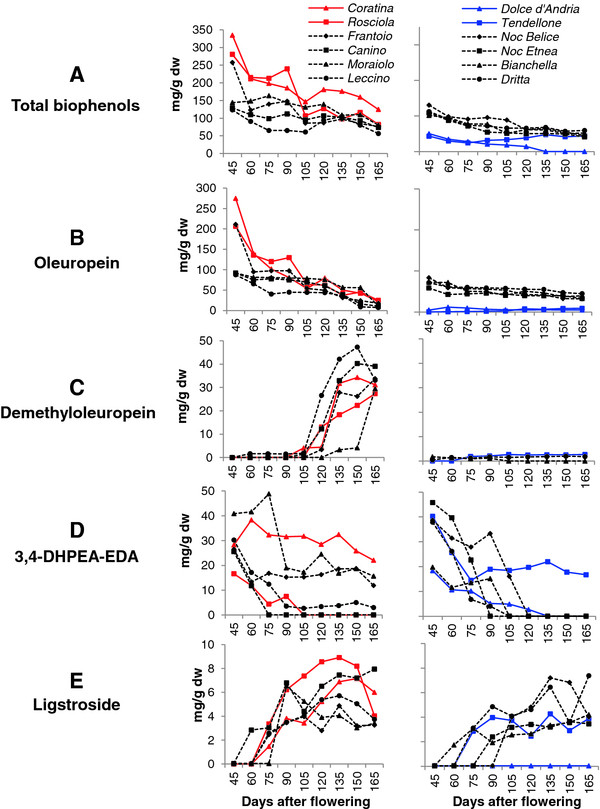
**Concentration of total phenols and secoiridoids compounds in olive fruits.** Phenolic compounds of 12 cultivars during fruit development (45, 60, 75, 90, 105, 120, 135, 150 and 165 DAF) were considered. **A**) Total biophenols, **B**) Oleuropein, **C**) Demethyloleuropein, **D**) 3,4-DHPEA-EDA, and **E**) Ligstroside. Demethyloleuropein was not detected in cvs. *Dolce d’Andria*, *Nocellara del Belice* and *Nocellara Etnea*. Red and Blue lines represent high (HP) and low phenolic (LP) cultivars, respectively. The standard errors are not shown in the graphs because the values were lower than 5%.

**Figure 2:**
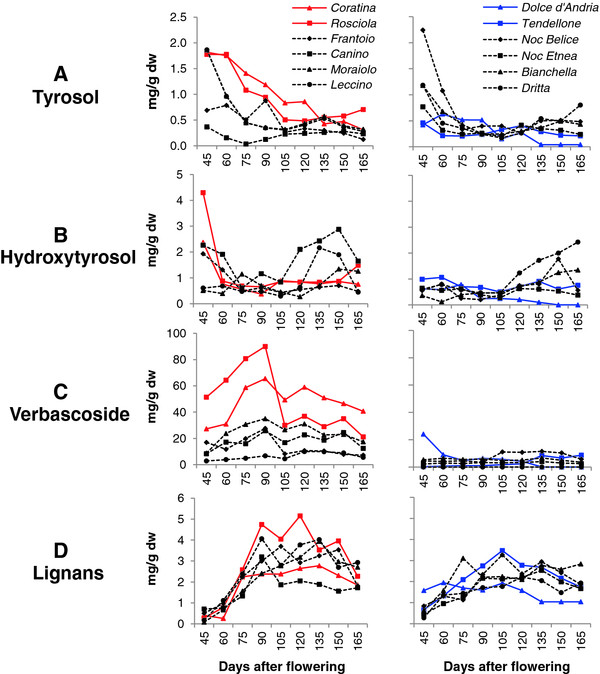
**Concentration of phenolic compounds in olive fruits.** Phenolic compounds of 12 cultivars during fruit growth (45, 60, 75, 90, 105, 120, 135, 150 and 165 DAF) were considered. **A**) Tyrosol, **B**) Hydroxytyrosol, **C**) Verbascoside, and **D**) Lignans. Red and Blue lines represent high (HP) and low phenolic (LP) cultivars, respectively. The standard errors are not shown in the graphs because the values were lower than 5%.

The total phenolic content decreased during fruit development, and at the first sampling time (45 DAF), it ranged from 50 to 350 mgg^-1^ dw in the 12 cultivars analysed (Figure 1A). The concentration of the major compounds, such as oleuropein, demethyloleuropein, 3–4 DHPEA-EDA, ligstroside, tyrosol, hydroxytyrosol, verbascoside and lignans, varied among cultivars, some of which had average phenolic levels higher than 150 mg g^-1^ dw (*Coratina* and *Rosciola*), while others had levels lower than 50 mg g^-1^ dw (*Tendellone* and *Dolce d’Andria*), and the levels in the remaining cultivars averaged from 60 to 130 mg g^-1^ dw (Figures 1 and 2, Additional file 1, Additional file 2 and Additional file 3).

In the high phenolics (HP) cultivars *Coratina* and *Rosciola*, oleuropein represented the most abundant compound (up to 82% of the total), decreasing in concentration after fruit set, as previously observed in other olive varieties [33]. In the low phenolics (LP) cultivars, *Tendellone* and *Dolce d’Andria*, the main phenolic compound was not oleuropein, lower than 15 mg g^-1^ dw during all stages of the maturation process (Figure 1B), but 3–4 DHPEA-EDA, which accounted for up to 94% of the total phenolic contents at the beginning of fruit development.

Demethyloleuropein showed an opposite trend, accumulating during the last period of fruit development, after 105 DAF, with the highest levels observed in the cvs. *Coratina, Rosciola, Frantoio, Canino, Moraiolo, * and *Leccino*. In contrast, only trace amounts of this compound (lower than 3 mg g^-1^ dw) were detected in *Tendellone, Bianchella, * and *Dritta*, and demethyloleuropein expression was completely absent in *Dolce d’Andria, Nocellara del Belice* and *Nocellara Etnea*, suggesting a block in the reaction leading to the synthesis or accumulation of this compound (Figure 1C). In HP cultivars the progressive decrease in the oleuropein concentration corresponded with an increase of demethyloleuropein content. The highest concentration of demethyloleuropein was observed at the stages with the lowest concentration of oleuropein. This observation supports the hypothesis of the derivation of demethyloleuropein from the demethylation of oleuropein, as previously envisaged by Obied *et al.*[1]. In addition, the observation that in LP cultivars and Nocellara del Belice, Nocellara Etnea, Bianchella and Dritta, oleuropein expression and demethyloleuropein content remain constant during fruit development is in accordance with this hypothesis.

The compound 3–4 DHPEA-EDA represented a consistent portion of the total phenolics during the early stages of fruit development in the cvs. *Tendellone, Dritta, Nocellara del Belice, * and *Nocellara Etnea, * (95%, 31.5% 29.7% and 42.3%, respectively), but the its concentration decreased to values nearing 0 at 90 DAF for most varieties (Figure 1D). Interestingly, in *Dolce d’Andria* it was the only secoiridoid detected at the early stages of fruit development, suggesting that in this cultivar secoiridoid synthesis is not totally blocked and also that this compound could be positioned upstream along the pathway before oleuropein, as reported by Ryan *et al.*[8].

In all cultivars expressing ligstroside, the concentration of this compound increased with fruit development (Figure 1E). This trend was opposite to that observed for oleuropein, suggesting that this compound might be derived from oleuropein rather than acting as its precursor.

Among phenolics, the tyrosol concentration showed a decreasing pattern from less than 2 mg g^-1^ dw, whereas the hydroxytyrosol concentration remained below 4 mg g^-1^ dw, with minor variation among cultivars and fruit developmental stages; notably, a slight increase in hydroxytyrosol concentration was observed after 120 DAF in *Canino*, *Leccino*, *Dritta* and *Bianchella* (Figure 2A-B, respectively).

Verbascoside, a molecule comprising tyrosol/hydroxytyrosol and cinnamyl-derivative moieties [1], was detected in consistent amounts (up to 90 mg g^-1^ dw) in *Coratina* and *Rosciola* at 90 DAF, while its concentration remained lower than 30 mg g^-1^ dw in the other cultivars during all developmental phases (Figure 2C), in accordance with the results of Jemai *et al.*[34]. These authors detected verbascoside only in earlier maturation stages in the cv. *Dhokar*. Based on our data, it seems that the two major phenolic compounds of olive fruits, oleuropein and verbascoside, with common tyrosol/hydroxytyrosol moieties, do not show any clear relationship.

The lignans acetoxypinoresinol and pinoresinol (Figure 2D) showed a modest increase in expression during fruit development, with the former being greater than the latter. The strongest difference in the relative content of these compounds was observed in *Dolce d’Andria*, for which the acetoxypinoresinol contents were approximately ten times higher than those of pinoresinol (Additional file 4).

The metabolic analysis allowed us to select HP and LP cultivars for further transcriptional analyses, as the contrasting phenotypes of these cultivars might reflect differences in the expression of the genes involved in phenolic metabolism.

### Identification of transcripts putatively involved in the synthesis of the main secondary metabolites identified from fruit EST datasets

**Figure 3:**
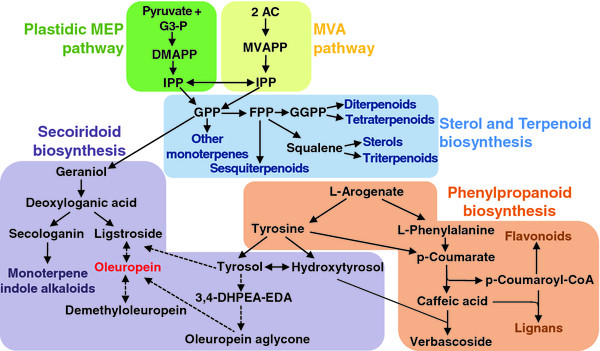
**Schematic representation showing the putative biosynthetic pathways of main secondary compounds of olive fruits.** G3P: glyceraldehyde 3-phosphate; DMAPP: Dimethylallyl diphosphate; IPP: Isopentenyl diphosphate; AC: Acetyl-CoA; MVAPP: Mevalonate diphosphate; GPP: Geranyl diphosphate; FPP: Farnesyl diphosphate; and GGPP: Geranyl geranyl pyrophosphate. Dotted arrows indicate uncertain biosynthetic steps.

A total of 27 transcripts (Table 1, Additional file 5) identified from the OLEA fruit EST database [30,35] were selected for their putative function within secondary metabolite pathways, considering the major compounds present in olive fruits, such as secoiridoids, phenolics, terpenes and sterols (Figure 3). Although the genes involved in the secoiridoid pathway are unknown because they are synthesised only in a restricted number of species whose genome sequence data are not yet available, it is still possible to predict some of the required enzymatic functions by observing proposed biosynthetic steps. Furthermore, many proposed reactions are similar to those working in parallel pathways in well-characterised plant species (e.g., the indole alkaloid pathway studied in *Catharanthus roseus*) [36-38]. 

**Table 1:** Transcripts putatively involved in the secondary metabolism of olive fruits

**Pathway**	**Transcript**	**Accession Number**	**Length (bp)**	**Enzymatic function**^**b**^	**Blast results**^**c**^
MEP pathway	OeDXS*	JX266162	574	1-deoxy-d-xylulose 5-phosphate synthase (EC:2.2.1.7)	7.9E-124, 92% *(H. brasiliensis)*
	*OeDXR*	JX266164	596	1-deoxy-d-xylulose-5-phosphate reductoisomerase (EC:1.1.1.267)	2.5E-129, 92% *(H. brasiliensis)*
	*OeCDPMES*	JX266166	927	2-C-methyl-D-erythritol 4-phosphate cytidyltransferase (EC:2.7.7.60)	4.0E-141, 87% *(S. miltiorrhiza)*
	*OeCDPMEK*	JX266168	800	4-diphosphocytidyl-2-C-methyl-D-erythritol kinase (EC:2.7.1.148)	5.1E-111, 76% *(S. miltiorrhiza)*
	*OeMECPS*	JX266170	550	2-C-methyl-D-erythritol 2,4-cyclodiphosphate synthase (EC:4.6.1.12)	5.8E-89, 75% *(A. adenophora)*
	*OeHMBPPS*	JZ030838	178	4-hydroxy-3-methylbut-2-en-1-yl diphosphate synthase-like (EC 1.17.7.1)	9.0E-32, 95% (*V. vinifera*)
	OeHMBPPR*	JX266172	1114	4-hydroxy-3-methylbut-2-enyl diphosphate reductase (EC:1.17.1.2)	0.0, 87% *(S. lycopersicum)*
	*OeIPPI*	JX266174	840	Isopentenyl diphosphate isomerase (EC:5.3.3.2)	2.3E-156, 95% *(N. tabacum)*
Mevalonate pathway (MVA)	*OeHMGR*	JZ030840	214	3-hydroxy-3-methyl glutaryl CoA reductase (EC:1.1.1.34)	7.6E-40, 89% *(S. nigrum)*
	*OeMVAK*	JX266176	1152	Mevalonate kinase (EC:2.7.1.36)	4.1E-142, 84% *(C. roseus)*
	*OeMVAPK*	JX266178	366	Phosphomevalonate kinase (EC:2.7.4.2)	6.2E-24, 67% *(V. vinifera)*
	*OeMVAPPD*	JX266179	373	Mevalonate diphosphate decarboxylase (EC:4.1.1.33)	1.0E-14, 85% (*C. roseus*)
Synthesis of monoterpenic moiety of secoiridoids	*OeGES*	JX266180	1090	geraniol synthase (EC:4.2.3.-)	4.0E-166, 76% (*P.dulcis*)
	*OeGE10H*	JX266182	1232	Geraniol 10-hydroxylase (EC:1.14.13.B15)	0.0, 80% *(C. roseus)*
	OeNDHD*	GQ851611	643	NADH dehydrogenase I (EC:1.6.99.3)	1.0E-92, 77% *(P. trichocarpa x P. deltoides)*
	OeGT*	GQ851612	1050	Glucosyltransferase (EC:2.4.1)	8.0E-126, 69% *(N. tabacum)*
	*OeSLS1*	JX266184	907	Secologanin synthase-like (EC:1.3.3.9)	1.2E-145, 67% *(V. vinifera)*
	*OeSLS2*	JX266186	1325	Secologanin synthase-like (EC:1.3.3.9)	1.1E-120, 45% *(G. max)*
	*OeSLS3*	JX266188	832	Secologanin synthase-like (EC:1.3.3.9)	1.8E-79, 44% *(G. max)*
	*OeSLS4*	JX266190	667	Secologanin synthase (EC:1.3.3.9)	1.3E-44, 38% *(C. roseus)*
	*OeLAMT*	JX266191	572	S-adenosylmethionine-dependent methyltransferase (EC:2.1.1.50)	1.9E-65, 53% *(V. vinifera)*
Synthesis of phenolic moieties	OeADH*	GQ851610	790	Arogenate dehydrogenase (EC:1.3.1.43)	6.0E-70, 84% *(S. pennellii)*
	OeCuAO*	GQ851613	1843	Copper amine oxidase (EC:1.4.3.21)	0.0, 89% *(R. communis)*
	*OePPO*	JX266193	1491	Polyphenol oxidase (EC:1.10.3.1)	0.0, 60% *(S. indicum)*
	*OeTYRD*	JX266195	1373	Tyrosine/dopa decarboxylase (EC:4.1.1.25)	0.0, 78% *(P. somniferum)*
	*OeALDH1*	JX266197	793	Alcohol dehydrogenase (EC:1.1.1.90)	2.0E-163, 98% *(O. europaea*)
	*OeALDH2*	JX266199	487	Alcohol dehydrogenase class-3 (EC:1.1.1.90)	1.0E-95, 93% *(G.max*)
Phenylpropanoid biosynthesis	*OePAL*	JX266200	1587	Phenylalanine ammonia-lyase (EC:4.3.1.24)	0.0, 90% *(P. frutescens)*
	*Oe4CL*	JX266202	260	4-coumarate coenzyme A ligase (EC: 6.2.1.12)	9.1E-50, 98% *(P. fortunei)*
Sterol and terpene biosynthesis	*OeLS*	JZ030839	169	Limonene synthase like (EC: 4.2.3.20)	6.0E-17, 67% *(A. thaliana*)
	*OeFPPS*	JX266204	1011	Farnesyl diphosphate synthase (EC:2.5.1.10)	0.0, 89% *(G. uralensis)*
	*OeSQS*	JX266206	516	Squalene synthase (EC: 2.5.1.21)	1.2E-111, 95% *(B. monnieri)*
	*OeGGPS*	JX266207	752	Geranylgeranyl pyrophosphate synthase (EC:2.5.1.29)	2.1E-159, 90% *(C. roseus)*
	*OeLUPS*^*d*^	AB025343^d^			
Degradation of phenolics	OeGLU*	HQ585436	857	Beta-1,3-glucosidase (EC:3.2.1.39)	6.0E-85, 85% *(R. communis)*
	OePOX*	GQ851609	1182	Peroxidase (EC: 1.11.1.7)	4.0E-140, 82% *(R. communis)*

^a^ The GenBank accession numbers of the transcripts are provided. Numbers JZ030838, JZ030840 and JZ030839 refer to the dbEST collection.

^b^ Putative enzymatic function and Enzyme Commission (EC) number are provided.

^c^ The E-value and the percentage identity from the best hit of the BLASTX search are provided. These values were used to indicate the significance of sequence similarity. The text in parentheses indicates the species of the best hit from BLASTX search.

^d^ Transcript sequenced and characterised by Shibuya *et al.*[28].

^*^ Transcripts derived from the cDNA-AFLP and RACE PCR analyses.

The unigenes showing best tBLASTn scores to protein sequences functionally characterised in other species were selected for subsequent transcriptional analyses. To restrict the number of candidates, only those with the highest levels of expression, deduced by the number of ESTs per contig, were considered. In fact, taking into account that phenolic compounds represent the most abundant secondary metabolites of olive fruit, we assumed that the metabolism should be strongly oriented to their biosynthesis. The E-value and the percentage identity to known proteins have been reported for the selected genes (Table 1, Additional file 5). Although this method has a certain level of uncertainty and further studies are necessary to confirm the proposed functions, the identification of candidate genes considering the similarity to known proteins is a powerful approach, as demonstrated for many other species.

The transcripts were resequenced for the cvs. *Coratina* and *Dolce d’Andria* and named according to their putative function.

Among the selected transcripts, some were involved in plastidial 2-C-methyl-d-erythritol 4-phosphate (MEP) and cytosolic mevalonate (MVA) pathways, whereas other candidate transcripts were putatively involved in the synthesis of secoiridoids (monoterpenic and phenolic moieties), other phenolics, terpenoids and sterols (Table 1).

Moreover, an olive gene encoding for a lupeol synthase (LUPS) [Genbank: AB025343], which resulted implicated in triterpenoid biosynthesis in yeast [28], was considered for the expression analyses.

### Identification of other transcripts from cDNA-AFLP analysis

To identify genes that might play a role in secondary metabolism but not included in the fruit EST dataset, a cDNA-AFLP analysis was performed. Comparing the fruit transcriptional profiles of the HP cvs. *Coratina* and *Rosciola* and LP cvs. *Tendellone* and *Dolce d’Andria* in three developmental stages (45, 90 and 165 DAF) allowed the identification of 93 fragments showing differential expression patterns. For 59 fragments, high quality sequences were obtained and blasted against protein databases. Forty-five transcripts (approximately 76% of selected sequences) showed significant similarity to proteins with known function. The BLAST results and expression patterns of these transcripts are shown in Additional file 6. The sequences of cDNA-AFLP fragments matched entries in the fruit EST database, with low differences attributable to cultivar allele variations, EST sequence uncertainties, or different genes of the same family.

The results of the Blast2GO analysis allowed the annotation of the expressed sequences according to the terms of three main Gene Ontology vocabularies. The majority of the transcripts encoded for putative proteins with binding or catalytic activities (44% and 37%, respectively) (Additional file 7).

For 24 transcripts, the expression profiles were confirmed using semi-quantitative PCR (sqPCR) analysis, and 23 transcripts showed a similar expression pattern to that observed in the cDNA-AFLP analysis (Additional file 8).

Seven transcripts (17,7%) were implicated in the synthesis or degradation of secoiridoids (Table 1). The sequences of the transcripts for *OeDXS*, *OeNDHD, OeGT*, *OeADH, OeCuAO*, *OeGLU* and *OePOX* were extended to 574, 643, 1050, 790, 1843, 857 and 1,182 bp, respectively, using RACE-PCR. The sequences of *OeNDHD* and *OePOX* include the complete coding and partial 5’ and 3’ UTR regions.

The *OeDXS* sequence exhibited high identity (86% identity, 92% similarity) to *DXS* type II [Genbank:CAD22531] of *M. truncatula*, which is putatively involved in secondary metabolism, and a lower BLASTP score (79% identity, 86% similarity) to *DXS* type I [Genbank:CAD22530], which is proposed to play a role in primary metabolism [39].

### Quantitative expression analyses for olive fruit mRNAs

The expression of the 28 candidate genes identified from EST datasets and the seven selected ones from the cDNA-AFLP analysis, putatively encoding for enzymes involved in secoiridoid, phenolic, terpene and sterol metabolism, was characterised using RT-qPCR to detect a possible correlation with the metabolic data.

The analysis was performed at 45, 90 and 165 DAF using the fruits of HP (*Coratina*) and LP (*Dolce d’Andria*) cultivars.

#### Relative expression of transcripts putatively involved in MEP and MVA pathways

In higher plants, the five-carbon building blocks of all terpenoids, isopentenyl diphosphate (IPP) and dimethylallyl diphosphate (DMAPP) may derive from the plastid-localised MEP pathway and the cytosolic MVA pathway. Enzymes and related genes in both pathways are well known and have been characterised.

**Figure 4:**
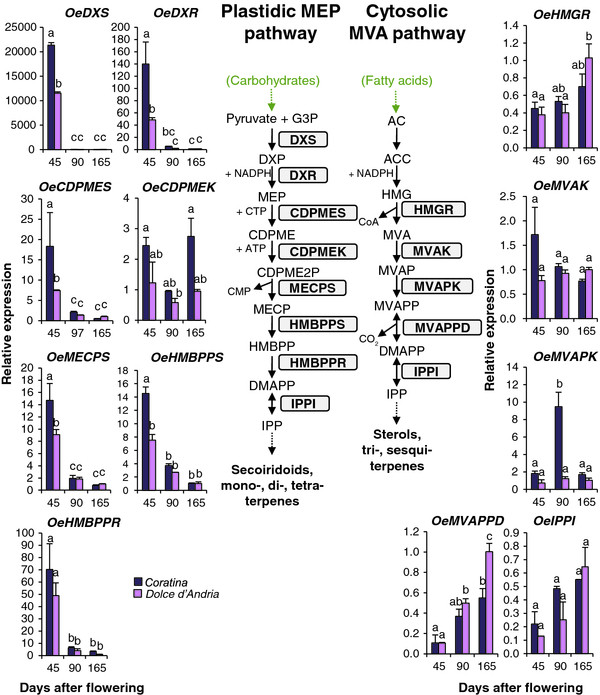
**The expression of genes putatively involved in the MEP and MVA pathways.** The mRNA expression of genes putatively involved in 2-C-methyl-d-erythritol 4-phosphate (MEP) and mevalonic acid (MVA) pathways, as determined using RT-qPCR and a schematic representation of these pathways. G3P: glyceraldehyde 3-phosphate; DXP: 1-deoxy-D-xylulose-5-P; DXS: DXP synthase; DXR: DXP reductoisomerase; MEP: 2-C-methyl-D-erythritol-4-P; CDPME: 4-(CDP)-2-C-methyl-D-erythritol; CDPMES: CDPME synthase; CDPMEK: CDPME kinase; CDPME2P: 4-(CDP)-2-C-methyl-D-erythritol-2-P; MECP: 2-C-methyl-D-erythritol 2,4-cyclo-PP; MECPS: MECP synthase; HMBPP: 1-hydroxy-2-methyl-2-(E)-butenyl-4-PP; HMBPPS: HMBPP synthase; HMBPPR: HMBPP reductase; DMAPP: Dimethylallyl diphosphate; IPP: Isopentenyl diphosphate; IPPI: IPP delta isomerase; AC: Acetyl-CoA; ACC: Acetoacetyl-CoA; HMG: 3-hydroxy-3-methylglutaryl-CoA; HMGR: HMGC reductase; MVAK: MVA kinase; MVAP: mevalonate phosphate; MVAPK: MVAP kinase; MVAPP: Mevalonate diphosphate; MVAPPD: MVAPP decarboxylase; and DAPP: Dimethylallyl diphosphate. The relative mRNA levels are expressed as ΔΔCt. Bars = ±SE, n = 3. Different letters indicate significant differences between samples, as determined using analysis of variance (Bonferroni’s post hoc tests, P < 0.05).

The six transcripts putatively involved in the MEP pathway (*OeDXSOeDXROeCDPMES, OeMECPS* and *OeHMBPPR* and *OeHMBPPS*) (Additional file 9) showed a strong significant decrease in expression from 45 DAF to subsequent developmental stages, whereas *OeCDPMEK* did not show a significant expression modulation (Figure 4). *OeDXSOeDXR* and *OeHMBPPR* had the strongest differential expression in accordance with their key role in the MEP pathway, which has been proposed in other plant species [40-42]. In particular, our results showed that *OeDXS,* which is involved in the first step of the MEP pathway, was exclusively expressed in the first developmental stage (45 DAF) in both cultivars. Based on current studies, there are two types of DXS. Type I is constitutively expressed in photosynthetic tissues and is likely involved in the biosynthesis of isoprenoids of primary metabolism, such as carotenoids and phytols, whereas type II DXS seems to be involved in the biosynthesis of isoprenoids for specialised metabolism [43]. It is possible that in the olive there is more than one DXS isoform, and the deduced amino acid sequence of our *OeDXS* showed similarity to *DXS* type II, suggesting its involvement in isoprenoids for secoiridoid biosynthesis rather than for primary metabolism. It has been reported that in grape a *DXS* gene co-localises with a major QTL, affecting monoterpene content [44], and it is putatively responsible for muscat flavour [45]. At 45 DAF, *OeDXSOeDXROeCDPMES, OeHMBPPR* were significantly more expressed in *Coratina* than in *Dolce d’Andria*. *OeIPPI*, coding for an enzyme involved in both the MEP and MVA pathways for the conversion of IPP to dimethylallyl pyrophosphate (DMAPP), did not show a significant expression difference among the samples (Figure 4). 

The four transcripts putatively encoding enzymes involved in the MVA pathway (*OeHMGR*, *OeMVAK*, *OeMVAPK*, *OeMVAPPD*) showed completely different profiles compared with the MEP transcripts. In particular, *OeHMGR* and *OeMVAK* did not display strong differences among the developmental stages or varieties, except for a weak expression increase at 165 DAF in cv. *Coratina* (Figure 4).

Two isoforms of the enzyme HMGR were identified in *Coffea arabica* fruits and transcripts of isoform *CaHMGR1* were expressed only at the initial stages of fruit development, while the isoform *CaHMGR2* was constitutively expressed [46]. It is also possible that these two isoforms are expressed in the olive fruit, and it is likely that we have identified the constitutive form.

*Coratina OeMVAPK* was more expressed at fruit pit hardening (90 DAF), whereas in *Dolce d’Andria,* it was expressed at the same level in all three developmental stages. *OeMVAPPD* mRNA increased during fruit development and was always more highly expressed (up to 13-fold) in *Coratina* than in *Dolce d’Andria*.

Unlike transcripts involved in the MVA pathway, the expression patterns of transcripts involved in the MEP pathway, in accordance with secoiridoid, decrease during fruit development, suggesting that the MEP pathway is also present in olives and might contribute to the terpenoid portion of secoiridoids. These results are consistent with those reported in other plant species, where MEP and MVA pathways produce different terpenoid classes. In fact, it is generally accepted that geranyl diphosphate (GDP) and geranylgeranyl diphosphate (GGDP), deriving from the MEP pathway, are used in plastids as substrates for monoterpene, diterpene and tetraterpene synthesis, whereas farnesyl diphosphate (FDP), obtained from the MVA pathway in the cytosol, is involved in triterpene and sesquiterpene biosynthesis [47]. However, cross talk between these two different IPP biosynthetic pathways has been documented, and the relative contribution of each pathway to the biosynthesis of the various classes of terpenes remains uncertain [48]. In olive, only a contrasting report has been published [49], suggesting the involvement of the MVA pathway in secoiridoid formation in the *Oleaceae* family, but neither enzymatic nor molecular data were provided.

#### Relative expression of transcripts putatively involved in biosynthesis of the terpenic moiety of secoiridoids

Biosynthetic steps leading to formation of the terpenic and phenolic portions of secoiridoids are still not well clarified and, as a consequence, enzymes involved in these pathways remain uncertain.

**Figure 5:**
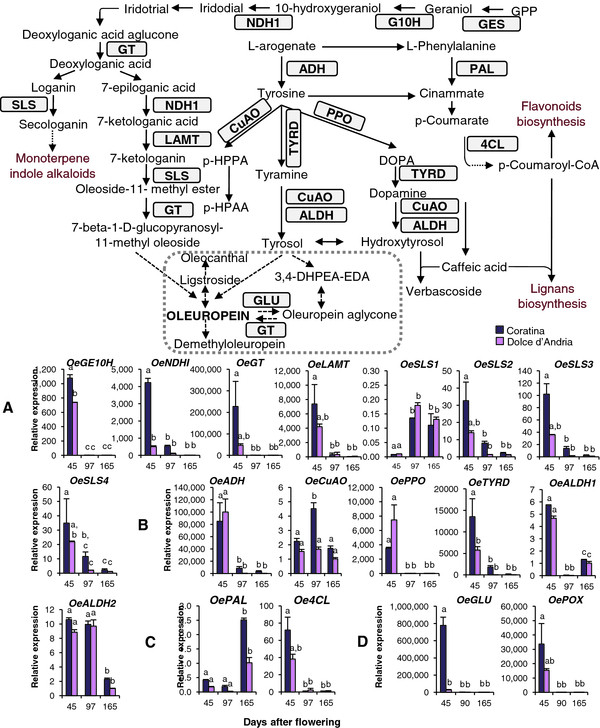
**The expression of genes putatively involved in the metabolism of secoiridoids and other phenolics compounds.** The mRNA expression of genes putatively involved in the biosynthesis of terpenic (**A**) and phenolic moieties (**B**) of secoiridoids, in the biosynthesis of phenylpropanoids (**C**), in the degradation of phenolic compounds, and a schematic representation of their metabolism. The mRNA level was determined using RT-qPCR. GES: geraniol synthase; G10H: Geraniol 10-hydroxylase; NDHI: NADH dehydrogenase I; GT: SLS: Secologanin synthase; LAMT: Loganic acid methyltransferase; ADH: Arogenate dehydrogenase; CuAO: Copper amine oxidase; *p*-HPPA: *p*-hydroxyphenylpyruvic acid; *p*-HPAA: *p*-hydroxyphenylacetic acid; TYRD: Tyrosine/dopa decarboxylase; ALDH: Alcohol dehydrogenase; PPO: Polyphenol oxidase; PAL: Phenylalanine ammonia-lyase; and 4CL: 4-coumarate coenzyme A ligase. The relative mRNA levels are expressed as ΔΔCt. Bars = ±SE, n = 3. Different letters indicate significant differences between samples as determined using analysis of variance (Bonferroni’s post hoc tests, P < 0.05). Grey dotted box includes the secoiridoids compounds. Dotted arrows indicates uncertain biosynthetic steps.

Transcripts putatively involved in the synthesis of the terpenic portion of secoiridoids (Additional file 10), *OeGES*, *OeGE10H*, *OeNDHD*, *OeGT*, *OeLAMT*, and the four transcripts putatively coding for secologanin synthase, *OeSLS1*, *OeSLS2*, *OeSLS3* and *OeSLS4*, were analysed (Figure 5A). Their expression dramatically decreased from 45 to 165 DAF, with the largest variation occurring between 45 and 90 DAF, in accordance with the decay of oleuropein concentration observed during fruit ripening. Significant differences between varieties were also observed for *OeGE10H* and *OeNDHI*, which appeared to be more highly expressed in *Coratina* than in *Dolce d’Andria* at 45 DAF.

GES is a monoterpene synthase involved in the synthesis of geraniol [50], and GE10H is a cytochrome P450 monooxygenase that hydroxylates the monoterpenoid geraniol at the C-10 position to generate 10-hydroxygeraniol. This enzyme was reported to be involved in the biosynthesis of iridoid monoterpenoids and several classes of monoterpenoid alkaloids observed in a diverse range of plant species [51]. Feeding experiments on *Syringa* and *Fraxinus* (other genera within the Oleaceae family) showed that the biosynthesis of oleoside-type secoiridoids proceeds via iridodial [25,26,52], which presumably originates from geraniol and 10-hydroxygeraniol [1,49,53] (Additional file 10). Interestingly, it has been recently shown using *in vitro* enzymatic assays that the same *OeGES1* that we have analysed is involved in the synthesis of geraniol [54], demonstrating the effectiveness of our approach in identifying candidate transcripts for phenolics biosynthesis.

The G10H of *C. roseus* (CrG10H) also catalyses the 3'-hydroxylation of naringenin to produce eriodictyol with a catalytic activity efficiency that is 10 times lower compared to geraniol hydroxylation. These studies demonstrated that G10H plays an important role in the biosynthesis of flavonoids, in addition to its previously described role in the metabolism of terpenoids [51]. Based on these studies, we cannot exclude that OeG10H might also be involved in flavonoid biosynthesis in the olive.

Despite the fact that NDHI and GT are enzymes required in different pathways, the strong differential expression of *OeNDHI* and *OeGT* in LP and HP varieties and during different stages of fruit development, suggested a putative role for these transcripts in secoiridoid metabolism. These enzymes might play a role in various steps of the pathway. NDHI might work in both, the conversion of iridotrial to deoxyloganic acid aglucone and the conversion of 7-epi-loganic acid to 7-ketologanic acid. GTs are required for terpenic and phenolic fractions, transferring glucosylic groups to deoxyloganic aglucones for the formation of deoxyloganic acid, the conversion of oleoside 11-methyl ester to 7-ß-1-D-glucopyranosyl 11-methyloleoside and catalysing the formation of oleuropein from oleuropein aglycone, which is the last step of the pathway (Additional file 10).

The position of 3,4-DHPEA-EDA along the secoiridoid pathway remains controversial. It has been considered either as a derivative of oleuropein, produced by its enzymatic degradation by endogenous β-glucosidases [14] (Additional file 2), or as the intermediate compound of an alternative biosynthetic pathway leading to oleuropein formation [1,8,55]. The high level of expression of genes encoding enzymes promoting the conversion of 3,4-DHPEA-EDA to oleuropein, such as *OeGT*, during the early stages of fruit development in the HP cv. *Coratina* supports the hypothesis of 3,4-DHPEA-EDA as an oleuropein precursor. This hypothesis finds a further confirmation in the observation that 3,4-DHPEA-EDA is the only secoiridoid detected in cv. *Dolce d’Andria* at the early stages of fruit development, suggesting that a downstream block may prevent the formation of the other secoiridoids.

In *O. europaea* both epoxides of secologanin and secoxyloganin could be precursors of oleuropein [49,53]. The oxidation required for the conversion of 7-ketologanin to oleoside-11- methyl ester is similar to the mechanism taking place for the conversion of loganin, an epimer of 7-ketologanin, to secologanin. Therefore, we assumed that the gene encoding this enzymatic function might have high sequence similarity with the secologanin synthase (a cytochrome P450 enzyme). Moreover, the role played by loganin and secologanin in secoiridoid biosynthesis in *Olea europaea* remains controversial, and it cannot be excluded that these compounds might be intermediates of secoiridoid biosynthesis [52], further supporting the involvement of a secologanin synthase.

In the fruit EST database, we identified four transcripts (*OeSLS1OeSLS2OeSLS3OeSLS4*) showing high similarities to secologanin synthase. Using RT-qPCR analyses, *OeSLS2OeSLS3OeSLS4* showed a pattern similar to the other transcripts that were included in the secoiridoid synthesis, supporting the hypothesis of their involvement in this pathway. However, *OeSLS1* showed a low level of expression at the first sampling, which increased later. This result could be explained by the putative involvement of *OeSLS1* in the biosynthesis of other secondary compounds, such as terpene indole alkaloids, as reported in other plant species [56], rather than playing a major role in oleuropein synthesis.

The methylation of 7-ketologanic acid might be catalysed by an enzyme similar to loganic acid O-methyltransferase (LAMT), which converts loganic acid to loganin (epimers of 7-ketologanic acid and 7-ketologanin, respectively), as indicated by the functional characterization in *C. roseus* (CrLAMT), showing that this enzyme exhibits high specificity for the loganic acid substrate [38]. Therefore, we identified a putative olive homolog (*OeLAMT*) of the *LAMT* gene.

#### Relative expression of transcripts putatively involved in phenolics biosynthesis

The phenolic moiety of secoiridoids is presumably derived from tyrosine and proceeds through tyrosol [1,8] (Additional file 11). In most plants, tyrosine is synthesised from arogenate decarboxylated by arogenate dehydrogenase (ADH) [57,58], and hydroxytyrosol is synthesised from tyrosine through DOPA and dopamine. Recently, the biosynthesis of hydroxytyrosol was clarified in *Olea europaea* using cultured cells [59]. Another pathway for the tyrosol formation has been reported in other plant species, where tyrosol might be produced from a *p*-coumaric acid precursor, which is derived primarily from phenylalanine [60]. However, in olive, the presence of this alternative pathway has never been demonstrated. Instead, two alternative routes from tyrosol to oleuropein have been proposed: one considering ligstroside as direct oleuropein precursor [49] and the other proceeding via oleuropein aglycone [8] (Additional file 11). Alternative biosynthetic pathways are proposed for verbascoside, e.g., from tyramine via dopamine or from tyrosol via hydroxytyrosol [59].

A schematic representation of the putative olive phenolics biosynthetic pathway is proposed in Figure 5, according to the previous findings discussed above. Based on these findings, we searched for transcripts putatively involved in the biosynthesis of the phenolic portion of secoiridoids and other related phenolic compounds, such as tyrosol, hydroxytyrosol and verbascoside.

We selected and analysed the following genes that might function in the considered pathway: *OeADHOeCuAOOePPOOeTYRDOeALDH1* and *OeALDH2*. ADH decarboxylates the arogenate producing tyrosine [61]. CuAO deaminates various compounds with biologically active amines producing their corresponding aminoaldehydes, H_2_O_2_ and NH_3_[62]. PPO catalyses the *o*-hydroxylation of monophenols to *o*-diphenols, and it might be involved in different steps of phenolics metabolism [63]. An enzyme similar to a tyrosine/dopa decarboxylase (TYRD) is required for both the conversion of tyrosine in tyramine and DOPA in dopamine [64,65]. The conversion of both tyramine to tyrosol and dopamine to hydroxytyrosol requires an amino-oxidase (AO) and an alcohol dehydrogenase (ALDH) [59,60].

The expression profiles of four of the six genes (*OeADH*, *OePPO, OeTYRD* and *OeALDH1*) correlated with the secoiridoid content, decreasing during fruit development, similarly to the genes involved in the terpenic moiety (Figure 5B). mRNAs of *OeADH*, *OePPO* and *OeTYRD* were exclusively present at 45 DAF, and *OeALDH1* and *OeALDH2* were more highly expressed at the first sampling and strongly decreased with fruit development. *OeTYRD* was more expressed in *Coratina* at 45 DAF compared to *Dolce d’Andria*. Only *OeCuAO* was more highly expressed at 90 DAF, decreasing at 165 DAF in *Coratina*, whereas *OeCuAO* expression remained constant during development in *Dolce d’Andria*.

In phenylpropanoid metabolism, two key genes have been investigated: *PAL* and *4CL*. They are involved in the synthesis of large groups of compounds, such as flavonoids, lignans and verbascoside. The expression of *OePAL* increased at 165 DAF in both cultivars, whereas *Oe4CL* showed the highest expression only at the first sampling in both cultivars (Figure 5C). The strong increase of *OePAL* mRNA levels at 165 DAF is consistent with the accumulation of anthocyanins and the change in fruit colour to purple-black that occurs in this phase [66]. The opposite trend observed for *Oe4CL* did not correlate with the content of lignans and flavonoids. In fact, the concentration of lignans first increased and then decreased during fruit growth in all cultivars, whereas the main flavonoids, rutin and luteolin 7-O-glucoside, are reported to increase during olive fruit ripening [67]. It is possible that *Oe4CL* might not be associated with lignans or flavonoid formation but with that of unique phenylpropanoid end-products, as reported for some species, such as Arabidopsis, aspen and soybean [68].

#### Relative expression of transcripts putatively involved in phenolics degradation

The expression of putative *OeGLU* and *OePOX* orthologs was analysed. GLU and POX enzymes are involved in phenolic degradation; moreover, GLU plays a role in the formation of oleuropein and ligstroside derivatives [5] (Additional file 2).

*OeGLU* was almost exclusively expressed during the early stages of fruit development (45 DAF) of cultivar *Coratina*, whereas *OePOX* was almost exclusively expressed at 45 DAF in both cultivars (Figure 5D). These profiles are similar to those observed for transcripts putatively involved in secoiridoid synthesis. These results confirm the role of these enzymes in processes that lead to the decrease in phenolic concentration observed at 90 and 165 DAF, and their expression might be down-regulated whenever a lower availability of oleuropein, their main substrate, occurs [69]. A similar mechanism might explain the lower expression observed for both types of transcripts in the LP cultivar, where the lack of oleuropein could be due to differences in the regulation of enzymes involved in its biosynthesis rather than in its degradation.

#### Relative expression of transcripts putatively involved in terpenoid and sterol biosynthesis

**Figure 6:**
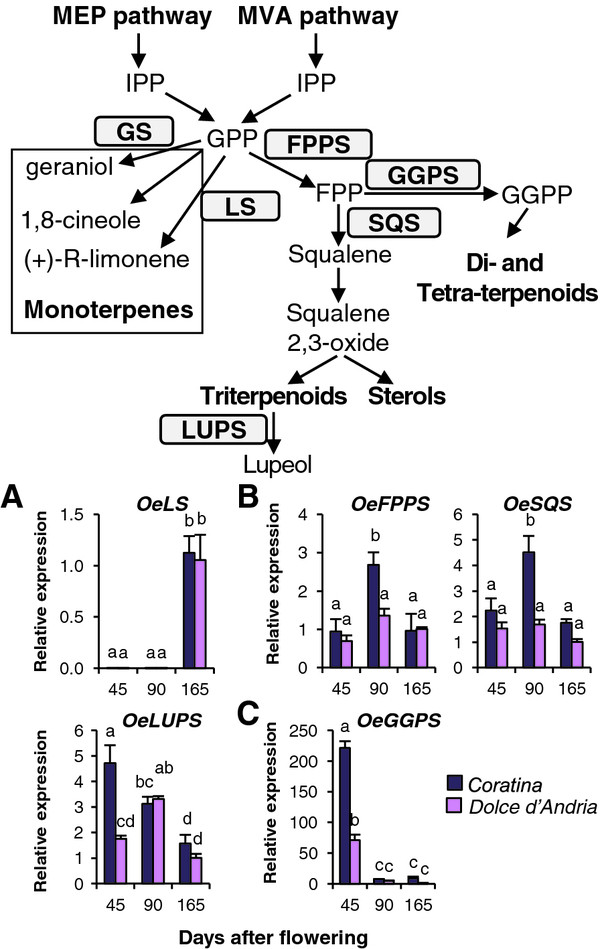
**The expression of transcripts putatively involved in the biosynthesis of terpenes and sterols.** The mRNA expression of genes putatively involved in the biosynthesis of volatile monoterpenes (**A**), tri-terpenoids and sterols (**B**), di- and tetra-terpenoids (**C**), as determined using RT-qPCR, and a schematic representation of this pathway. IPP: Isopentenyl diphosphate; GPP: Geranyl diphosphate; GES: geraniol synthase; LS: Limonene synthase; FPP: Farnesyl diphosphate; FPPS: FPP synthase; GGPP: Geranyl geranyl pyrophosphate; GGPS: GGPP synthase; SQS: Squalene synthase; LUPS: Lupeol synthase. The relative mRNA levels are expressed as ΔΔCt. Bars = ±SE, n = 3. Different letters indicate significant differences between samples as determined using analysis of variance (Bonferroni’s post hoc tests, P < 0.05).

The expression of *OeLS*, putatively leading to the synthesis of limonene, an important volatile monoterpene, showed a strong variation during fruit development. In particular, it was almost exclusively detected at the last sampling in both analysed cultivars (Figure 6A). This pattern is perfectly consistent with the limonene content in unripe and ripe fruits [70]. 

Oe*FPPS* and *OeSQS* mRNA levels, putatively coding for two key enzymes leading to the synthesis of squalene, were also analysed, and their expression reached a peak at 90 DAF in *Coratina* (Figure 6B). It is noteworthy that important compounds are synthesised from squalene-oxide at the onset of fruit development, such as maslinic and oleanolic acids and α- and β-amyrins [13]. The expression profiles observed for *OeFPPS* and *OeSQS* of *Coratina* were consistent with these findings. In contrast, differences among developmental stages were not observed in *Dolce d’Andria*.

The expression profile in developing fruits of the gene for lupeol synthase, catalysing the formation of triterpenic lupeol and functionally characterised in yeast [28], has never been analysed. Our *OeLUPS* showed a different mRNA profile between the two cultivars at 45 DAF (Figure 6B), which was more highly expressed in *Coratina* than in *Dolce d’Andria*, and, in the last two stages, they shared the same pattern. These results could be explained by a different accumulation of squalene and triterpenes during fruit development in *Dolce d’Andria*; however, the accumulation of these compounds in this variety has not yet been investigated.

The mRNA levels of GGPS, catalysing the synthesis of geranyl geranyl pyrophosphate (GGPP), an important intermediate for diterpenes and carotenoids formation, were higher at 45 DAF compared with the other developmental stages in both cultivars, and, at the same stage, the expression was higher in *Coratina* (Figure 6C). Our results are consistent with the higher accumulation of carotenoids during the first stages of olive fruit development [71].

## Conclusions

Our study represents an effort to characterise the transcriptional profile of candidate genes putatively involved in secondary metabolism in olive fruits by assessing the content of major phenolic compounds in a comprehensive number of cultivars at different fruit developmental stages. These methods allowed us to identify candidate genes for secondary metabolites.

We observed a strong variation in overall metabolite content, in the level of specific compounds and in the accumulation trends among genotypes and fruit developmental stages. *Coratina* and *Rosciola* were characterised by the highest oleuropein concentration at 45 DAF. In contrast, *Dolce d’Andria* and *Tendellone* showed the lowest content at all stages. Interestingly, we identified genotypes characterised by the complete absence of other compounds. In particular, *Nocellara del Belice*, *Dolce d’Andria* and *Nocellara Etnea* did not accumulate demethyloleuropein, and *Dolce d’Andria* was the only variety showing undetectable amounts of all secoiridoids, excepting 3,4-DHPEA-EDA at the early stages of fruit development. These findings might result from a block in the enzymatic steps leading to the biosynthesis of these compounds.

The evidence of an accumulation of ligstroside occurring when oleuropein decreases, leaves space to the hypothesis that ligstroside may derive from oleuropein instead of the contrary.

The observed differences in the phenolic profiles among the cultivars might reflect great variability in the modulation of their biosynthesis and accumulation. This variability can be exploited in breeding programmes to increase the fruit composition of important phenolic compounds.

The recent development of EST datasets for olive fruits allowed the extrapolation of gene information using sequence-similarity-based approaches. We used protein sequences that were previously characterised or had an assigned function in other species. The high level of similarity detected allowed us to predict the enzyme classes and, with some degree of approximation, the substrate specificity. As a complementary approach, the cDNA-AFLP analysis proved to be an efficient technique for the isolation of differentially expressed transcripts without any sequence similarity assumption.

Based on these approaches, 36 transcripts were identified and their expression profiles were characterised and associated with corresponding metabolite profiles.

The strong correlation observed between the content of specific metabolites during fruit development and the expression of transcripts putatively involved in their biosynthesis, suggests that metabolite content is regulated at transcriptional level and strengthens the involvement of the candidate genes in the proposed pathways. The differences observed in the expression of some genes between *Coratina* and *Dolce d’Andria* might indicate a different regulation of the transcripts involved in the secondary metabolism among olive genotypes.

In particular, the levels of most of transcripts putatively involved in the biosynthesis of secoiridoids (both terpenic and phenolic moieties) showed a strong decrease during fruit development, according to the decrease in oleuropein concentration at the same stages, and many genes involved in secoiridoid pathway were more highly expressed in HP cv. *Coratina* than in LP cv. *Dolce d’Andria*.

We observed a correlation between the MEP pathway and secoiridoid transcriptional profiles, supporting the hypothesis that this pathway, rather than the MVA pathway, primarily contributes to secoiridoid biosynthesis.

Interestingly, some key genes of monoterpenoid and phenolic biosynthesis, such as *OeDXS*, *OeGES*, *OeG10H* and *OeADH*, were exclusively expressed in the earliest sampling, when the highest secoiridoid concentration was detected. These genes might regulate the accumulation of these compounds during fruit development.

A strong correlation between metabolic and transcriptional data was also identified for the biosynthesis of limonene and GGPP. *OeLS* was almost exclusively expressed at the developmental stage when the highest level of this compound was detected. *OeGGPPS*, putatively involved in carotenoid biosynthesis, was expressed only in young fruits when the highest levels of these compounds occur, as reported in other studies.

These data provide useful information for functional genetic studies of this crop species and for the identification of functional markers related to the accumulation of compounds and metabolites affecting the nutraceutical and organoleptic properties of olive fruits and oil.

## Methods

### Plant material

Based on previous information on the phenolic profile of their oils, the following cultivars were chosen, putatively representing a high level of variation in fruit phenolics content: *Bianchella*, *Canino*, *Coratina*, *Dolce d’Andria*, *Dritta*, *Frantoio*, *Leccino*, *Moraiolo*, *Nocellara del Belice*, *Nocellara Etnea*, *Rosciola* and *Tendellone*. Fruits used for the phenolic composition and cDNA-AFLP analyses were harvested from 45 to 165 days after full bloom (DAF) every 15 days from plants of an olive cultivar collection at the experimental farm of the CRA–OLI (Collececco, Spoleto, Perugia) in central Italy (42° 48’ 48”N, 12° 39’ 15”E, 356 m above sea level). The phenological stage of the fruits at sampling dates was recorded. Plants were grown under the same environmental and agronomical conditions. To avoid possible effects of different levels of water availability on the phenolic content among trees, their water status was periodically monitored and occasional irrigation was applied as needed to maintain all plants at similar values of pre-dawn water potential during the sampling period (data not shown). Immediately after harvesting, the olive fruits were frozen in liquid nitrogen and stored at −80°C until further analysis.

### Evaluation of phenolic compounds

The extraction of phenolic compounds was performed according to Servili *et al.*[72], with few modifications, Briefly, 3 g of olive fruit mesocarp and exocarp were homogenised using a Homogeniser A/S N (Foss Electric, Denmark) in a 100-ml solution of methanol/water 80:20%, followed by two further homogenisation in 50 ml of methanol/water 80:20 using an Ultra-Turrax T 25 (IKA, Staufen, Germany). After methanol evaporation in vacuum under a nitrogen flow at 37°C, solid-phase extraction (SPE) was performed to separate the phenolics from the aqueous extract. During the SPE, a 900-mg Extraclean high load C_18_ cartridge (Alltech Italia s.r.l., Sedriano, Italy) was loaded with 1 ml of olive extract using 50 ml of methanol as the eluting solvent.

The HPLC analysis was performed according to Selvaggini *et al.*[73], using a Spherisorb ODS-1 250 mm x 4.6 mm column with a particle size of 5 *μ*m (Phase Separation Ltd., Deeside, UK).

The phenolic separation was performed using semi-preparative high-performance liquid chromatography (HPLC) analysis with a 9.4 mm i.d. 500-mm Whatman Partisil 10 ODS-2 semipreparative column; the mobile phase was 0.2% acetic acid in water (pH 3.1) (A)/methanol (B) at a flow rate of 6.5 mL/min and phenol detection was performed using a diode array detector (DAD). The purity of all compounds obtained from direct extraction was tested using HPLC, and their chemical structure was verified using nuclear magnetic resonance (NMR) with the same operative conditions reported in previous studies [72]. The following phenolic compounds were considered: oleuropein, demethyloleuropein, 3,4-DHPEA-EDA, ligstroside, tyrosol, hydroxytyrosol, verbascoside and lignans.

### Identification of mRNAs putatively involved in the metabolism of phenolic compounds

Sequences derived from the OLEA EST database [35], a collection of over 102,000 *Olea europaea* L. fruit EST reads generated through 454 massive sequencing technology [30], were analysed to identify transcripts putatively involved in the pathways of terpenoids, phenolics and other secondary metabolite synthesis. Amino acid sequences of genes involved in the pathways of interest and published in the Kyoto Encyclopaedia of Genes and Genomes (KEGG) databases [74,75] were used to search olive homologs in the OLEA EST database using basic local alignment (tBLASTn). For those genes represented using multiple unique transcripts, only the largest contig and/or that representing the highest number of singletons was used for the analysis. Sequences obtained from this collection include candidates for the isoprenoid (both MVP and MEP pathways), phenylpropanoid, terpene (monoterpenes, secoiridoids, diterpenes and triterpenes), phenol, sterol, lignan and flavonoid biosynthesis, leading to the main secondary metabolites present in the olive fruit. All transcripts of interest were resequenced in the HP and LP cultivars, *Coratina* and *Dolce D’Andria*, respectively, using gene-specific primers (Additional file 12).

### cDNA-AFLP analysis

Total RNA was isolated from fruit mesocarp and exocarp using the RNeasy Plant Mini Kit (Qiagen), and contaminating genomic DNA were removed with DNase I (Qiagen) treatment. Samples of two HP (*Coratina* and *Rosciola*) and two LP (*Tendellone* and *Dolce d’Andria*) cultivars, at three samplings (45, 90 and 165 DAF), were considered.

PolyA RNA was isolated from approximately 50 μg of total RNA using oligo dT Dynabeads (Invitrogen). All purified mRNA was used to synthesise first strand cDNA using SuperScript III (Invitrogen) according to the manufacturer’s 18-bp oligo d(T) protocol. Double-stranded (ds) cDNA was synthesised by incubating the first-strand product for 2 h at 16°C with 30 U of DNA polymerase I (Invitrogen) and 3 U of RNase H (Invitrogen) in a reaction mixture containing 20 mM Tris–HCl, 75 mM KCl, 10 mM (NH_4_)_2_SO_4_, 5 mM MgCl_2_ and 1 mM DTT. Subsequently, a 10-μl aliquot of each sample was assessed on a 1% agarose gel, and a clear DNA smear was visible between 500 and 4,000 bp. The samples were purified using a phenol/chloroform procedure and quantified using a spectrophotometer at a wavelength of 260 nm.

The cDNA-AFLP procedure was conducted according to Bachem and coworkers [76], with some modifications. A total of 500 ng of cDNA was digested in a 50-μl volume using 10 U of *Mse*I and 20 U of *Eco*RI for 2 h at 37°C. The digestion mix was ligated to 5 pmol of *Eco*RI adapter and 50 pmol of *Mse*I adapter using 68 U of T4 Ligase (New England Biolabs). The reaction was performed for 2 h at 37°C. The PCR reaction solution (50 μl) for preamplification contained 10 μl of the digestion mix, 75 ng of each primer constructed using the adaptors, 0.2 mM dNTP mix, 1.5 mM Mg^2+^ and 1 U of Taq polymerase (Invitrogen). The PCR reaction was conducted using the following conditions: 94°C for 2 min, 25 cycles at 94°C for 30 sec, 56°C for 1 min and 72°C for 1 min, followed by 72°C for 7 min. The selective amplification was performed using 5 μl of preamplification product diluted 1:10, 60 ng of each selective primer, 1.5 mM Mg^2+^, 0.2 mM dNTP mix and 1 U of Taq polymerase in a 20 μl total reaction volume. A total of 20 combinations of the selective primers containing 3 and 2 selective bases for the primer were designed using the *Eco*RI and *Mse*I adapters, respectively, and the Eco primers were labelled with fluorescein. Touch-down PCR was performed using the following cycling parameters: 94°C for 2 min, 13 cycles at: 94°C for 30 sec, 65°C (−0.7°C/cycle) for 30 sec and 72°C for 1 min, 23 cycles at 94°C for 30 sec, 56°C for 30 sec and 72°C for 1 min, followed by 72°C for 7 min. The selected PCR product (5.7 μl) was denatured at 96°C for 12 min and separated on a 4% denatured polyacrylamide gel, pre-run at 6 Watts for 10 min and run at 125 Watts for 1.75 h at 50°C in Genomyx GX 100 (Beckman Instruments, Fullerton, CA). The gel was scanned, and the separated fragments were visualised on a gel using fluorescent emission. Any differentially expressed fragment was excised from the gel, treated in a speed vacuum for 30 sec and incubated in a 100 μl volume of water overnight at 4°C. A total of 5 μl of each sample was reamplified with 50 ng of the same primers used in the preamplification procedure, 1.5 mM Mg^2+^, 0.2 mM dNTP mix and 1 U of Taq polymerase in a 20 μl total reaction volume. The PCR reaction was conducted using the following conditions: 94°C for 2 min; 35 cycles at: 94°C for 30 sec, 56°C for 1 min and 72°C for 1 min, followed by 72°C for 7 min. A 5 μl aliquot of the PCR mixture was run on an agarose gel, and the remaining reactions were purified with PolyEthylene Glycol (PEG) 8000. The cDNA fragments were eluted in 10 μl of H_2_O and either directly sequenced or sequenced after subcloning into the pGEM-T Easy Vector (Promega) followed by plasmid purification with QIAprep Spin Miniprep kit (Qiagen). The fragments were sequenced using the automated sequencer 3130 Genetic Analyser (Applied Biosystems, Foster City, CA).

The sequences were aligned to the non-redundant protein sequences (nr) database at the NCBI [77] using BLASTX, which also calculated protein similarity and predicted the hypothetical function of the unknown fragment sequences.

All sequences differentially expressed in cDNA-AFLP analysis were annotated according to the three main gene ontological categories (cellular component, biological process and molecular function) using the Blast2GO software v1.3.3 [78,79] according to Galla and coworkers [31], with minor modifications. All sequences were also aligned to the OLEA EST database using the BlastN algorithm to detect possible homologous transcripts.

Enzyme mapping of the annotated sequences was performed using a direct GO to Enzyme annotation, and this programme was also used to query the KEGG maps to define the main metabolic pathways involved. The conserved domains were identified using the Conserved Domain Database (CDD) [80,81].

Sequences showing expression profiles compatible with the content of corresponding metabolites were further characterised. Fragments were extended at 3’ and 5’ ends using a RACE-PCR method to obtain long partial or full-length cDNA clones. The resulting sequences were used to confirm the putative function of each compound. RACE-PCR was performed using the SMART RACE cDNA Amplification Kit (Clontech), with some modifications. Briefly, total RNA (2 μg) was reverse transcribed using the 3’CDS primer II A and the SMART II A oligonucleotide (Clontech). The reactions were incubated in a solution containing 50 mM Tris–HCl pH 8.3, 75 mM KCl, 6 mM MgCl_2_ and 2 mM DTT for 1.5 h at 42°C with Superscript III (Invitrogen). For the second strand synthesis, touch-down PCR was performed using a small aliquot (1/20 volume) of the primary template with EX Taq (Takara), the 5’ PCR primer II A (Clontech) and a gene specific primer. A thermal cycling programme was performed using the following conditions: an initial denaturation step of 94°C for 2 min, followed by 16 cycles of 94°C for 30 sec, 68°C for 30 sec with a 0.5°C decrease per cycle and 72°C for 3 min. An additional 20 cycles were performed at 94°C for 30 sec, 60°C for 30 sec and 72°C for 3 min, with a final elongation step at 72°C for 5 min. Nested PCR was required in some cases to improve the specificity of the amplification. The samples were purified using PEG 8000 and either directly sequenced or sequenced after being cloned into the pGEM-T Easy vector with the 3130 Genetic Analyser (Applied Biosystems, Foster City, CA).

### Semiquantitative RT-PCR

To validate the differential expression patterns of cDNA-AFLP selected clones, preliminary semi-quantitative PCR experiments were performed on 24 transcripts using different biological replicates of the samples. The RNeasy Plant Mini Kit (Qiagen) was used to isolate 2 μg of total RNA from biological replicates of the olive samples used in the cDNA-AFLP analysis. The cDNA synthesis was performed using SuperScript III according to the manufacturer’s protocol. Elongation Factor (*EF1α*) was used as the endogenous gene for sample normalisation [31]. Specific *EF1α* primers were used for cDNA sample normalisation with the following conditions: a 25 μl total reaction volume containing 10 μl of first strand cDNA diluted 1:50, 1.5 mM Mg^2+^, 0.2 mM dNTP mix and 1.25 U of Taq polymerase (Invitrogen). The following PCR amplification conditions were used: 94°C for 3 min, 20 to 45 cycles (depending on the amplicon signal intensity obtained under non-saturating PCR conditions) at 94°C for 30 sec, 60°C for 30 sec and 72°C for 50 sec, followed by 72°C for 7 min. The cDNAs were normalised based upon band intensity. After sample normalisation, a PCR was performed using the previously described conditions with 16-bp specific primers that included the restriction site sequences (*Eco*I and *Mse*I) used for the cDNA-AFLP analysis and a fragment specific region (Additional file 13) to validate both the expression and the allelic polymorphisms of any genes of interest.

### Quantitative RT-qPCR

The RT-qPCR experiments were performed on the most representative candidates of main branches of secondary metabolites maps. RT-qPCR was performed on the subset of most robust 37 putative transcripts to verify if their expression profiles corresponded to the patterns of secoiridoid, phenolics and other secondary metabolites synthesis or degradation. *Coratina* and *Dolce d’Andria* were used as reference HP and LP varieties, respectively. Total RNA was extracted from 0.2 g of fruit mesocarp and exocarp with the RNeasy Plant Mini Kit (Qiagen) and treated with DNase I (Qiagen). Reverse transcription of 2 μg of RNA was performed using oligo(dT)_18_ and the SuperScript III Reverse Transcriptase kit (Invitrogen) according to the manufacturer’s instructions. Quantitative real-time PCR was performed on a PCR Real Time 7300 (Applied Biosystems, Foster City, CA) according to the manufacturer's protocol and using the Reagent kit for SYBR Green analysis (Applied Biosystems) and gene-specific primers (Additional file 14). Primers were verified by the presence of a single PCR product band after agarose gel electrophoresis. All reactions were performed in triplicate. After each assay, a dissociation kinetics analysis was performed to verify the specificity of the amplification products. Relative amounts of all mRNAs were calculated using the 2^-ΔΔCT^ method [82], where ΔCt = Ct_target gene_ - Ct_reference gene_ using *Dolce D’Andria* at 165 DAF as a control sample. The housekeeping Elongation Factor 1-α gene was used as an endogenous reference gene for cDNA normalisation. The data for three biological replicates were analysed using an analysis of variance (ANOVA) followed by Bonferroni’s post hoc test (P < 0.05) with R software (version 2.14.0) [83].

## Abbreviations

DAF,Days after flowering; HP,High phenolics; LP,Low phenolics; cvs,Cultivars; RT-qPCR,Real-time reverse transcription polymerase chain reaction; G3P,Glyceraldehyde 3-phosphate; DXP,1-deoxy-D-xylulose-5-P; DXS,1-deoxy-D-xylulose-5-P synthase; DXR,1-deoxy-D-xylulose-5-P reductoisomerase; MEP,2-C-methyl-D-erythritol-4-P; CDPME,4-(CDP)-2-C-methyl-D-erythritol; CDPMES,2-C-methyl-D-erythritol 4-phosphate cytidyltransferase; CDPMEK,4-(CDP)-2-C-methyl-D-erythritol kinase; CDPME2P,4-(CDP)-2-C-methyl-D-erythritol-2-P; MECP,2-C-methyl-D-erythritol 2,4-cyclo-PP; MECPS,2-C-methyl-D-erythritol 2,4-cyclodiphosphate synthase; HMBPP,1-hydroxy-2-methyl-2-(E)-butenyl-4-PP; HMBPPS,1-hydroxy-2-methyl-2-(E)-butenyl-4-PP synthase; HMBPPR,1-hydroxy-2-methyl-2-(E)-butenyl-4-PP reductase; DMAPP,Dimethylallyl diphosphate; IPP,Isopentenyl diphosphate; IPPI,Isopentenyl diphosphate delta isomerase; AC,Acetyl-CoA; ACC,Acetoacetyl-CoA; HMG,3-hydroxy-3-methylglutaryl-CoA; HMGR,HMGC reductase; MVAK,MVA kinase; MVAP,Mevalonate phosphate; MVAPK,MVAP kinase; MVAPP,Mevalonate diphosphate; MVAPPD,Mevalonate diphosphate decarboxylase; DAPP,Dimethylallyl diphosphate; GES,Geraniol synthase; G10H,Geraniol 10-hydroxylase; NDH1,NADH dehydrogenase I; GT,Glucosyltransferase; SLS,Secologanin synthase; LAMT,Loganic acid methyltransferase; ADH,Arogenate dehydrogenase; CuAO,Copper amine oxidase; *p*-HPPA,*p*-hydroxyphenylpyruvic acid; *p*-HPAA,*p*-hydroxyphenylacetic acid; TYRD,Tyrosine/dopa decarboxylase; ALDH,Alcohol dehydrogenase; PPO,Polyphenol oxidase; PAL,Phenylalanine ammonia-lyase; 4CL,4-coumarate coenzyme A ligase; GPP,Geranyl diphosphate; LS,Limonene synthase; FPP,Farnesyl diphosphate; FPPS,Farnesyl diphosphate synthase; GGPP,Geranyl geranyl pyrophosphate; GGPS,Geranyl geranyl pyrophosphate synthase; SQS,Squalene synthase; LUPS,Lupeol synthase.

## Competing interests

The authors declare that they have no competing interests.

## Authors’ contributions

LB, MS, FA conceived the research plan. SC, SU, GV, SE, AT performed the analyses of olive phenolic compounds by HPLC. AR and SC monitored and controlled tree water status of plants. FA performed cDNA-AFLP analyses. FA and FP performed the identification of candidate genes from EST data. FA and RM performed the RT-qPCR experiments and the re-sequencing of candidate genes. FP and FA performed the statistical analyses on RT-qPCR data. FA and LB drafted the manuscript. FP, GP, MS, AR and RR critically revised the manuscript. All authors read and approved the manuscript.

## Supplementary Material

Additional file 1 **Chemical structures of main phenolic compounds of olive fruits.** Secoiridoid glucosides (oleuropein, demethyloleuropein, ligstroside) and verbascoside (hydroxycinnamic derivative observed in olive fruits) (from Servili *et al.*[5]). Click here for file

Additional file 2 **Putative biochemical mechanism of secoiridoid derivatives formation.** Figure from Servili *et al.*[5]. Click here for file

Additional file 3 Mean concentration of total phenolics in mesocarp and exocarp of olive fruits during fruit development. Click here for file

Additional file 4 **Pinoresinol and acetoxypinoresinol content in olive fruits.** Pinoresinol (A) and acetoxypinoresinol (B) contents in the 12 cultivars during fruit ripening (45, 60, 75, 90, 105, 120, 135, 150 and 165 DAF) were considered. Red and blue lines represent high (HP) and low phenolic (LP) cultivars, respectively. Standard errors are not shown in the graphs because these values were lower than 5%. Click here for file

Additional file 5 Transcripts selected from OLEA EST database. Click here for file

Additional file 6 **Transcript-derived fragments (TDFs) obtained by cDNA-AFLP analysis.** List of the fragments showing similarity to known proteins, their putative functions, Genbank accession numbers, their expression trends and homologues in OLEA database. Click here for file

Additional file 7 **GO terms distribution.** GO terms distribution in the biological processes (A), molecular functions (B) and cellular components (C) vocabularies. Click here for file

Additional file 8 **Comparison between cDNA-AFLP (A) and SQ-PCR (B) revealed the same expression pattern.** Some examples are reported for LP (*Dolce d’Andria* and *Tendellone*) and HP (*Coratina* and *Rosciola*) cultivars at three stages of fruit ripening (1, 2, 3 correspond to 45, 90, 165 DAF, respectively). Elongation Factor 1α was used as a reference gene (C). The putative heat shock proteins purple acid phosphatase, 1,3-β-glucosidase, ferredoxin chloroplast precursor, polygalacturonase, and 4-hydroxy-3-methylbut-2-enyl diphosphate reductase are reported. Click here for file

Additional file 9 **Biosynthesis of isoprenic unit of secoiridoids.** The MEP pathway for isoprenoid biosynthesis is reported. Through DXP synthesis and reduction, MEP is obtained and converted to CDPME through the transfer of a phosphocytidyl moiety. CDPME is phosphorylated to CDPMEP and cyclised to MECP. After an oxidoreduction reaction CDPMES is reduced to HMBPP, which is finally converted to IPP or DMAPP. Click here for file

Additional file 10 **Biosynthesis of terpenic moiety of secoiridoids.** The biosynthetic steps for the production of the terpenic moiety of oleuropein in *Olea europaea* according to Obied *et al.*[1] is reported, with some modifications. Based on our data, we propose that in the olive fruits geraniol for the secoiridoid synthesis derives from the MEP pathway. Iridoidal is produced through a series of hydroxylation and oxidation reactions on geraniol followed by a cyclisation reaction. Further oxidation yields iridotrial and deoxyloganic acid aglycone. Deoxyloganic acid is converted to 7-*epi*-loganic acid through the hydroxylation of the cyclopentane ring, then the hydroxyl group is oxidised to form 7-ketologanic acid. An esterification reaction is required for the conversion of 7-ketologanic acid to 7-ketologanin, and subsequently, oleoside 11-methyl ester is produced through the oxidation of the ketonic group. In a reaction catalysed by glucosyl transferase, oleoside 11-methyl ester is converted to 7-β-1-D-glucopyranosyl 11-methyl oleoside, which is the precursor of ligstroside and oleuropein. Click here for file

Additional file 11 **Biosynthesis of phenolic moiety of secoiridoids.** The biosynthetic pathway for the production of oleuropein and 3,4-DHPEA-EDA in *Olea europaea,* according to Ryan *et al.*[8], is reported. First *p*-hydroxyphenylalanine (tyrosine) is deaminated and oxidised by the enzyme amine oxidase to form *p*-hydroxyphenylpyruvic acid. Subsequently, *p*-hydroxyphenylacetic acid is generated through the decarboxylation of *p*-hydroxyphenylpyruvic acid. By reduction of *p*-hydroxyphenylacetic acid, *p*-hydroxyphenylethanol (tyrosol) is formed, and through a series of condensation reactions with oleoside, this product produces ligstroside, 3,4-DHPEA-EDA, and oleuropein. Click here for file

Additional file 12 **Primers used for the amplification of transcripts involved in secondary metabolite synthesis in olive.** Primer sequences and amplicon size are provided. Click here for file

Additional file 13 **Primers used for RT-sqPCR analyses.** Primer sequences and amplicon sizes are provided. Click here for file

Additional file 14 **Primers used for RT-qPCR analyses.** Primer sequences and amplicon sizes are provided. Click here for file
