# Supplementary figures and images for: Olive phenolic compounds: metabolic and transcriptional profiling during fruit development
Source: BMC Plant Biol. 2012 Sep 10;12:162. doi: 10.1186/1471-2229-12-162 (PMC3480905; doi:10.1186/1471-2229-12-162)

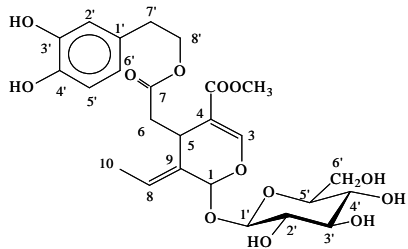

Oleuropein

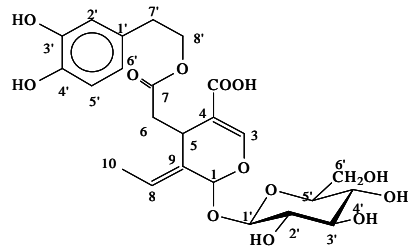

Demethyloleuropein

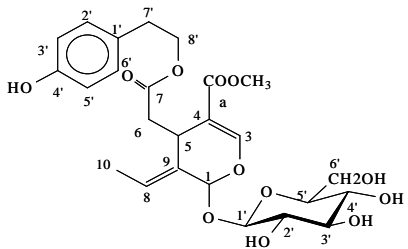

Ligstroside

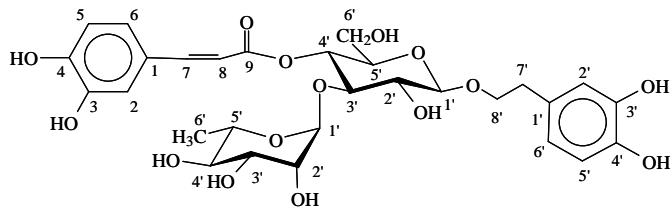

Verbascoside

Supplement: Additional file 1 — Chemical structures of main phenolic compounds of olive fruits. Secoiridoid glucosides (oleuropein, demethyloleuropein, ligstroside) and verbascoside (hydroxycinnamic derivative observed in olive fruits) (from Servili et al.[5]). [file 1471-2229-12-162-S1.pdf]

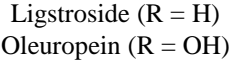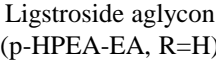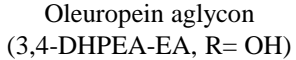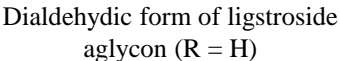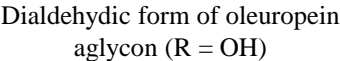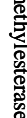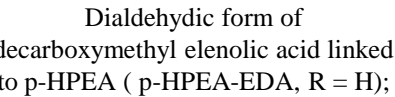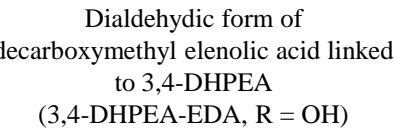

Supplement: Additional file 2 — Putative biochemical mechanism of secoiridoid derivatives formation. Figure from Servili et al.[5]. [file 1471-2229-12-162-S2.pdf]

# A

## Acetoxypinoresinol

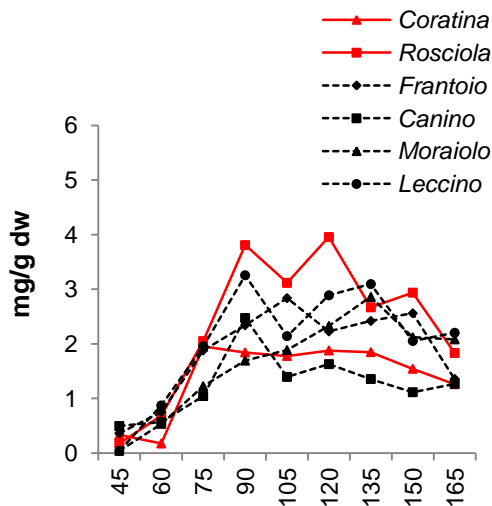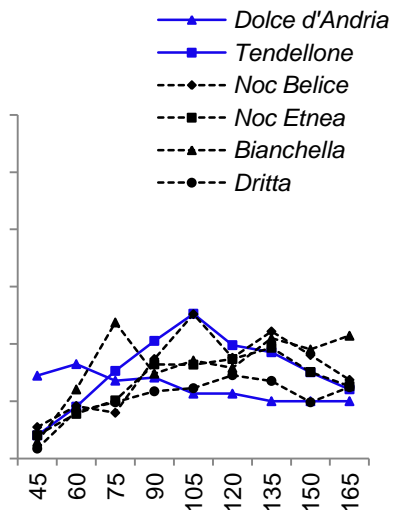

# B

## Pinoresinol

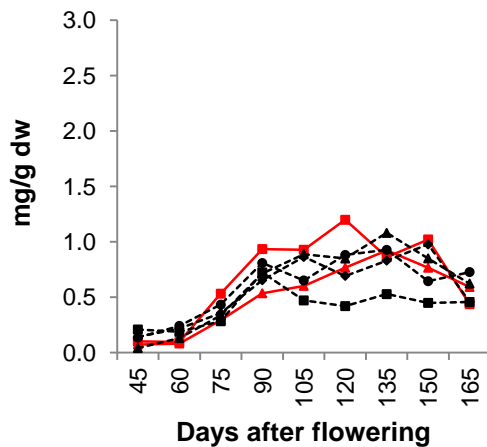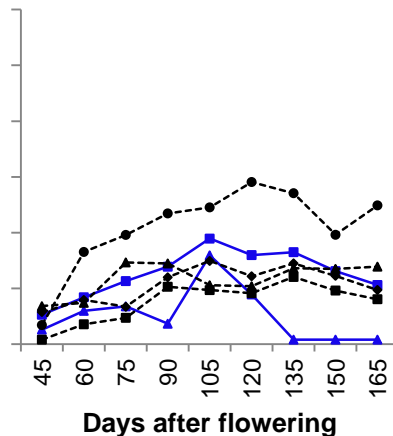

Supplement: Additional file 4 — Pinoresinol and acetoxypinoresinol content in olive fruits. Pinoresinol (A) and acetoxypinoresinol (B) contents in the 12 cultivars during fruit ripening (45, 60, 75, 90, 105, 120, 135, 150 and 165 DAF) were considered. Red and blue lines represent high (HP) and low phenolic (LP) cultivars, respectively. Standard errors are not shown in the graphs because these values were lower than 5%. [file 1471-2229-12-162-S4.pdf]

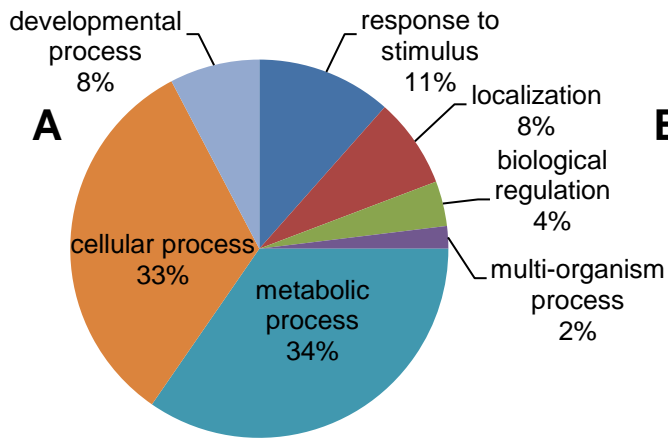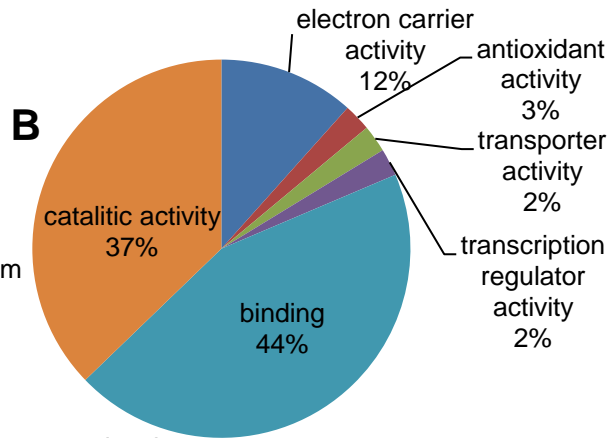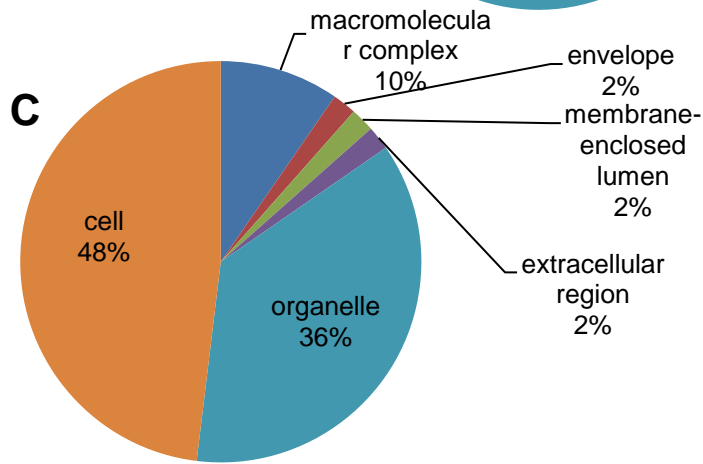

Supplement: Additional file 7 — GO terms distribution. GO terms distribution in the biological processes (A), molecular functions (B) and cellular components (C) vocabularies. [file 1471-2229-12-162-S7.pdf]

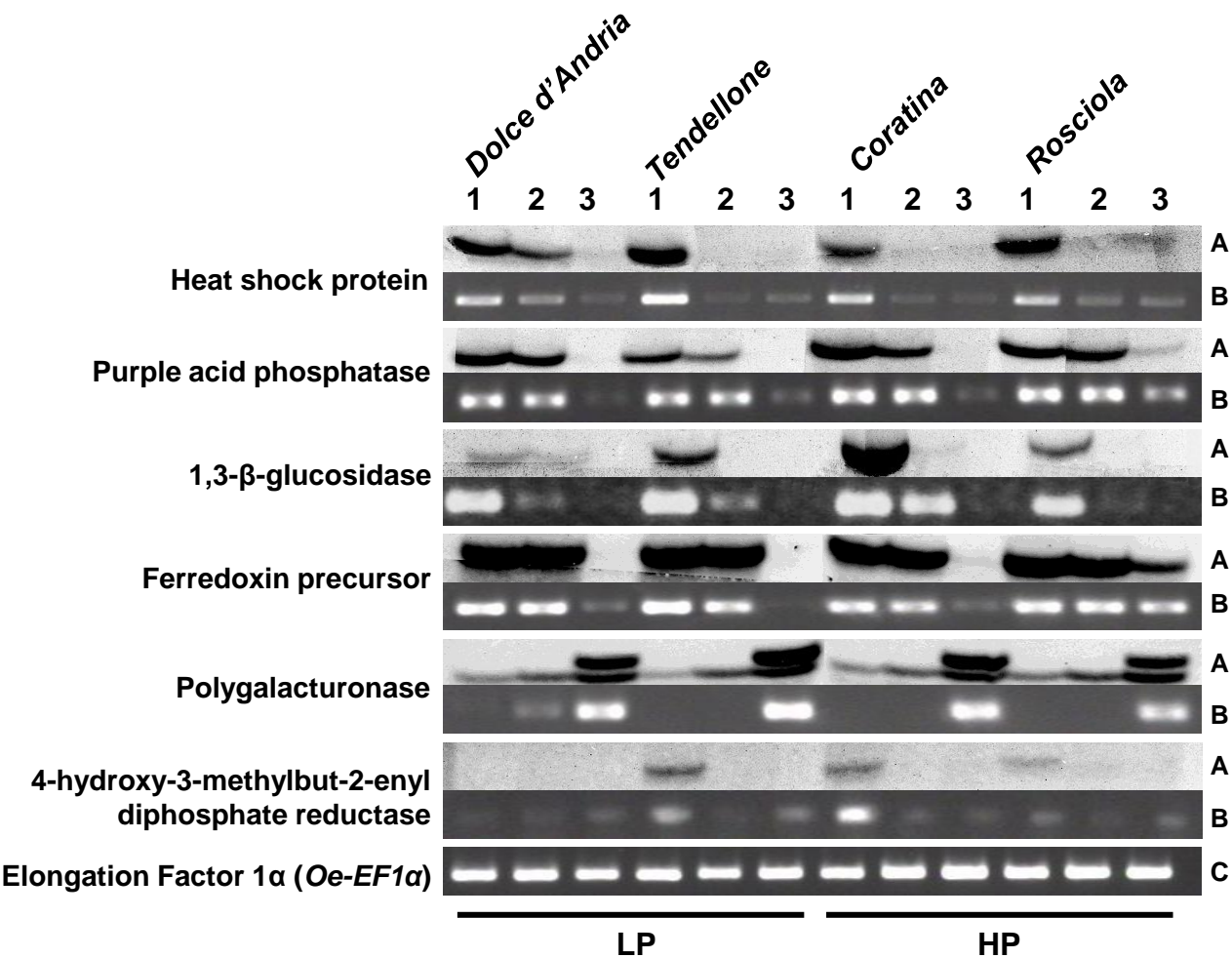

Supplement: Additional file 8 — Comparison between cDNA-AFLP (A) and SQ-PCR (B) revealed the same expression pattern. Some examples are reported for LP (Dolce d’Andria and Tendellone) and HP (Coratina and Rosciola) cultivars at three stages of fruit ripening (1, 2, 3 correspond to 45, 90, 165 DAF, respectively). Elongation Factor 1α was used as a reference gene (C). The putative heat shock proteins purple acid phosphatase, 1,3-β-glucosidase, ferredoxin chloroplast precursor, polygalacturonase, and 4-hydroxy-3-methylbut-2-enyl diphosphate reductase are reported. [file 1471-2229-12-162-S8.pdf]

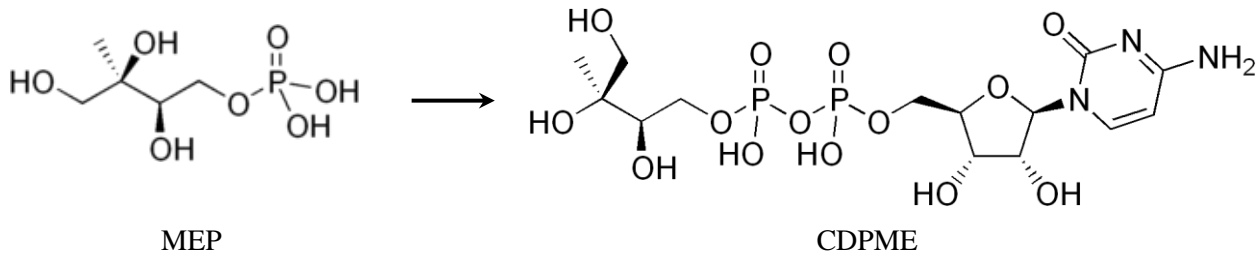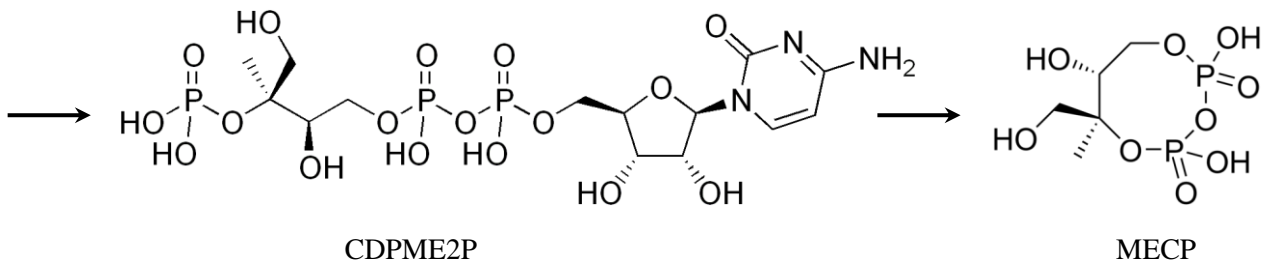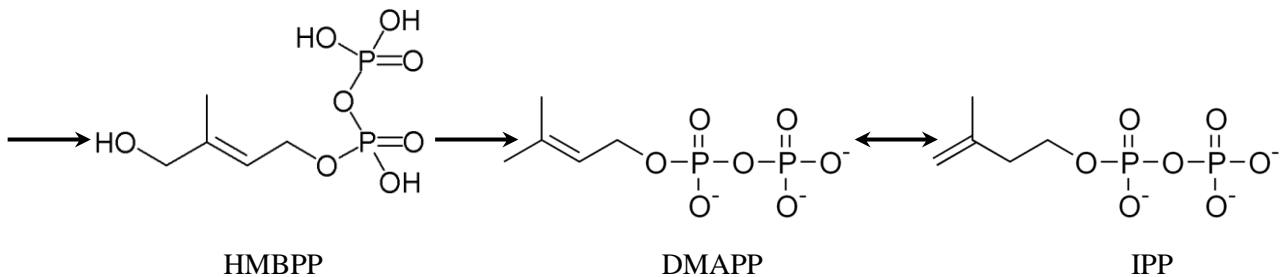

Supplement: Additional file 9 — Biosynthesis of isoprenic unit of secoiridoids. The MEP pathway for isoprenoid biosynthesis is reported. Through DXP synthesis and reduction, MEP is obtained and converted to CDPME through the transfer of a phosphocytidyl moiety. CDPME is phosphorylated to CDPMEP and cyclised to MECP. After an oxidoreduction reaction CDPMES is reduced to HMBPP, which is finally converted to IPP or DMAPP. [file 1471-2229-12-162-S9.pdf]

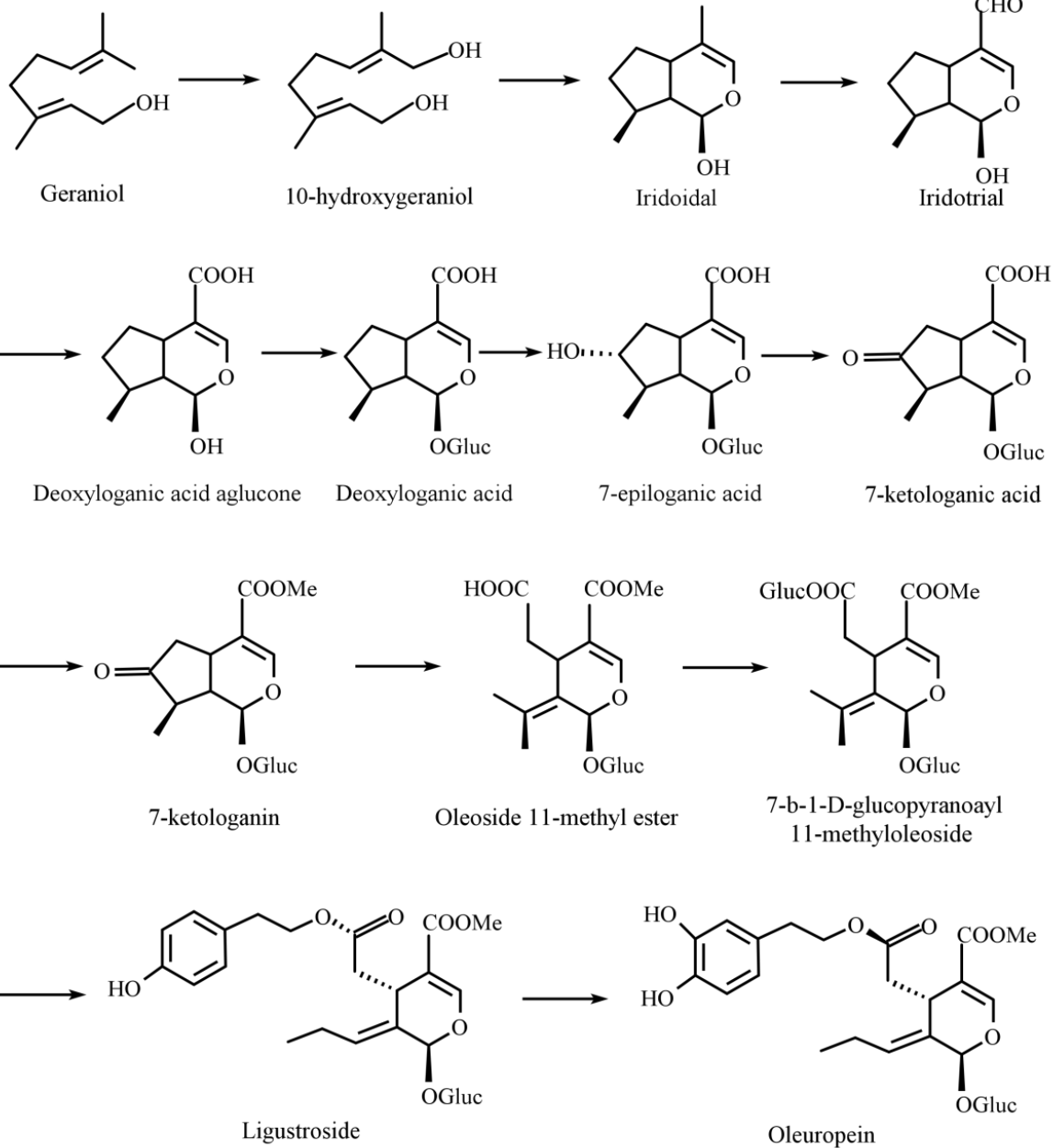

Supplement: Additional file 10 — Biosynthesis of terpenic moiety of secoiridoids. The biosynthetic steps for the production of the terpenic moiety of oleuropein in Olea europaea according to Obied et al.[1] is reported, with some modifications. Based on our data, we propose that in the olive fruits geraniol for the secoiridoid synthesis derives from the MEP pathway. Iridoidal is produced through a series of hydroxylation and oxidation reactions on geraniol followed by a cyclisation reaction. Further oxidation yields iridotrial and deoxyloganic acid aglycone. Deoxyloganic acid is converted to 7-epi-loganic acid through the hydroxylation of the cyclopentane ring, then the hydroxyl group is oxidised to form 7-ketologanic acid. An esterification reaction is required for the conversion of 7-ketologanic acid to 7-ketologanin, and subsequently, oleoside 11-methyl ester is produced through the oxidation of the ketonic group. In a reaction catalysed by glucosyl transferase, oleoside 11-methyl ester is converted to 7-β-1-D-glucopyranosyl 11-methyl oleoside, which is the precursor of ligstroside and oleuropein. [file 1471-2229-12-162-S10.pdf]

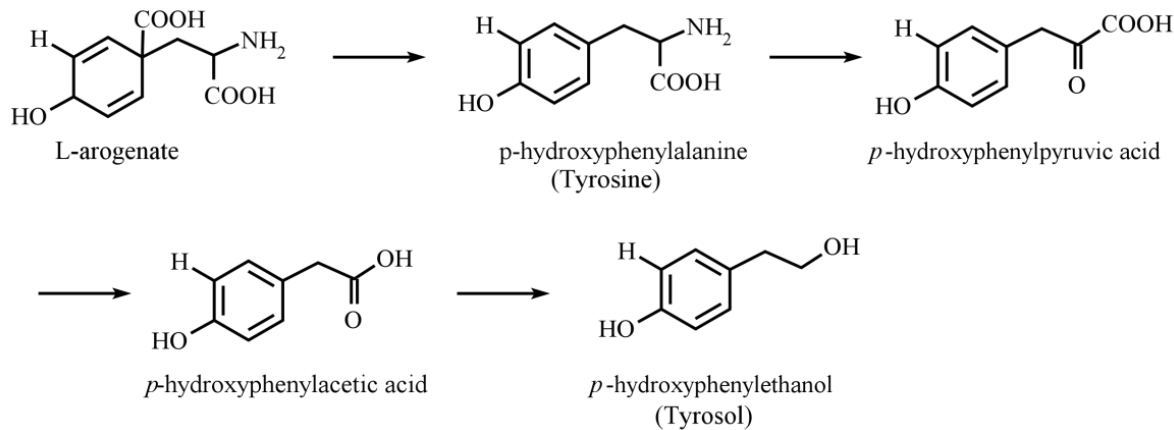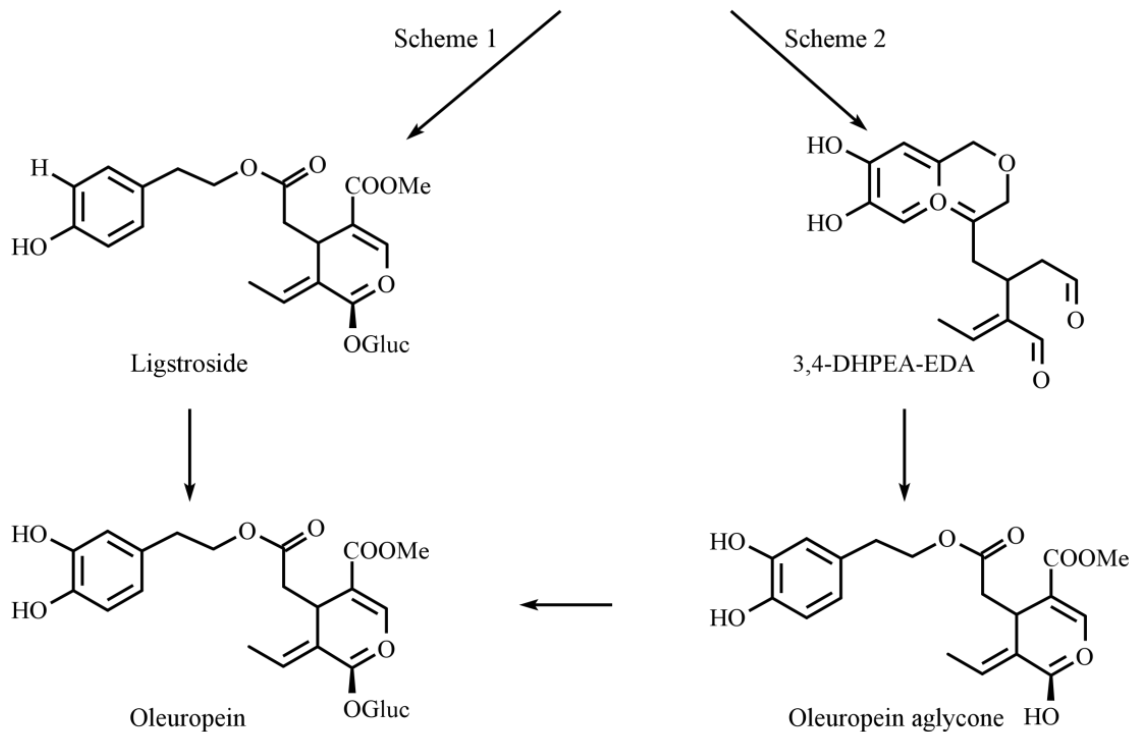

Supplement: Additional file 11 — Biosynthesis of phenolic moiety of secoiridoids. The biosynthetic pathway for the production of oleuropein and 3,4-DHPEA-EDA in Olea europaea, according to Ryan et al.[8], is reported. First p-hydroxyphenylalanine (tyrosine) is deaminated and oxidised by the enzyme amine oxidase to form p-hydroxyphenylpyruvic acid. Subsequently, p-hydroxyphenylacetic acid is generated through the decarboxylation of p-hydroxyphenylpyruvic acid. By reduction of p-hydroxyphenylacetic acid, p-hydroxyphenylethanol (tyrosol) is formed, and through a series of condensation reactions with oleoside, this product produces ligstroside, 3,4-DHPEA-EDA, and oleuropein. [file 1471-2229-12-162-S11.pdf]
