# Supplementary material for: Olive phenolic compounds: metabolic and transcriptional profiling during fruit development
Source: BMC Plant Biol. 2012 Sep 10;12:162. doi: 10.1186/1471-2229-12-162 (PMC3480905; doi:10.1186/1471-2229-12-162)
Supplement: Additional file 3 — Mean concentration of total phenolics in mesocarp and exocarp of olive fruits during fruit development. [file 1471-2229-12-162-S3.pdf]

Additional file 3 - Mean concentration of total phenolics in mesocarp and exocarp of olive fruits along fruit development.

Cultivar	Mean values o f total phenolics (mg g^{-1} dw)
Coratina	175.0±2.8
Rosciola	164.4±1.6
Moraiolo	130.0±1.3
Frantoio	107.0±2.0
Canino	102.3±1.4
Leccino	82.3±0.8
Nocellara del Belice	81.0±1.5
Dritta	73.8±0.8
Bianchella	69.7±0.7
Nocellara Etnea	62.8±0.8
Tendellone	37.0±1.1
Dolce d'Andria	18.4±1.5
