# Supplementary material for: Olive phenolic compounds: metabolic and transcriptional profiling during fruit development
Source: BMC Plant Biol. 2012 Sep 10;12:162. doi: 10.1186/1471-2229-12-162 (PMC3480905; doi:10.1186/1471-2229-12-162)
Supplement: Additional file 5 — Transcripts selected from OLEA EST database. [file 1471-2229-12-162-S5.pdf]

Additional file 5 –Transcripts selected from OLEA EST database

**Pathway**	**Transcript**	**OLEA EST DB ID[a]/ length (bp)/ N° of transcripts per contig**
	OeDXR	OLEEUCl006848:Contig2/750/35
	OeCDPMES	OLEEUCl007282:Contig1/595/10; OLEEUCl007282:Contig2/294/3
	OeCDPMEK	OLEEUCl008168:Contig1/1275/22
	OeMECPS	OLEEUCl051892:Contig1/256/2; OLEEUCl016737:Contig1/384/5
	OeHMBPPS	OLEEUCl003184:Contig1/1853/93
	OeIPPI	OLEEUCl019015:Contig2/1165/72
Mevalonate pathway (MVA)	*OeHMGR*	OLEEUCl026514:Contig1/245/2
	OeMVAK	OLEEUCl070212:Contig1/357/3
	OeMVAPK	OLEEUCl021472:Contig1/437/5
	OeMVAPPD	OLEEUCl036011:Contig2/1000/37
Synthesis of monoterpenic moiety of secoiridoids	*OeGES*	OLEEUCl011546:Contig1/1479/54
	OeGE10H	OLEEUCl013653:Contig2/1747/122
	OeSLS1	OLEEUCl030419:Contig1/1156/37
	OeSLS2	OLEEUCl002698:Contig1/904/64; OLEEUCl002698:Contig2/888/29
	OeSLS3	OLEEUCl005527:Contig1/1306/26
	OeSLS4	OLEEUCl002698:Contig2/888/29
	OeLAMT	OLEEUCl012404:Contig3/799/55
	OePPO	OLEEUCl061995:Contig1/ 342/2; OLEEUCl017472:Contig1/530/7
	OeTYRD	OLEEUCl001996:Contig2/1995/464
	OeALDH1	OLEEUCl010817:Contig1/1469/239
	OeALDH2	OLEEUCl045078:Contig1/725/18
Phenylpropanoid biosynthesis	*OePAL*	OLEEUCl011172:Contig1/1046/26
	Oe4CL	OLEEUCl051455:Contig1/564/7
Sterol and terpene biosynthesis	*OeLS*	E8NTSAO02DPRK9/233/1
	OeFPPS	OLEEUCl008872:Contig1/1318/83
	OeSQS	OLEEUCl036893:Contig1/643/8
	OeGGPS	OLEEUCl010559:Contig1/419/3

[a] Identification number (ID) of the homologue (EST or cluster) in OLEA database (http://140.164.45.140/oleaestdb/).
