# Supplementary material for: Olive phenolic compounds: metabolic and transcriptional profiling during fruit development
Source: BMC Plant Biol. 2012 Sep 10;12:162. doi: 10.1186/1471-2229-12-162 (PMC3480905; doi:10.1186/1471-2229-12-162)
Supplement: Additional file 6 — Transcript-derived fragments (TDFs) obtained by cDNA-AFLP analysis. List of the fragments showing similarity to known proteins, their putative functions, Genbank accession numbers, their expression trends and homologues in OLEA database. [file 1471-2229-12-162-S6.pdf]

Additional file 6 - Transcript-derived fragments (TDFs) obtained by cDNA-AFLP analysis

#TDF	Size (bp)	Acc. Num.[a]	Gene homology[b]	E-Value[c]	OLEA-EST DB ID[d]	Expression trend[e]
L1*	275	JK784410	**Peroxidase** *(Ricinus communis);* EEF31220	2.00E-36	E8NTSAO03GJ9PQ	HP <
L3*	317	JK784411	**4-hydroxy-3-methylbut-2-enyl diphosphate reductase** *(Vitis vinifera);* CBI32545	1.34E-13	OLEEUCl002062:Contig2	HP <
L5*	200	JK784412	Pectinesterase like protein *(Populus trichocarpa);* XP_002310162	5.80E-01	OLEEUCl062061:Contig1	HP <
L8*	284	JK784413	Zinc finger (dnl type) family protein *(Ricinus communis);* XP_002510604	6.05E-22	E8NTSAO02D4RGL	LP =
L13*	226	JK784414	Lipoxygenase *(Solanum lycopersicum);* AAA53184	1.00E-28	OLEEUCl026482:Contig1	HP >
M1*	500	JK784439	Pheophorbide A oxygenase *(Ricinus communis);* XP_002533389	1.48E-44	OLEEUCl057548:Contig1	<
M2	380	JK784440	**Glucosyl transferase** *(Nicotiana tabacum);* BAB88935	2.00E-30	OLEEUCl011437:Contig1	HP <
M3	268	JK784441	**NADH dehydrogenase I subunit m** *(Populus trichocarpa)* ; ABK96611	1.00E-41	-	<
M5	522	JK784442	Polygalacturonase *(Vitis vinifera);* CBI34471	1.01E-60	OLEEUCl040962:Contig1	LP <
M7*	317	JK784443	**Arogenate dehydrogenase** *(Populus trichocarpa);* XP_002331058	8.00E-48	E8NTSAO03GWKCZ	HP <
M9	179	JK784444	Reticuline oxidase precursor *(Ricinus communis);* EEF39195	1.00E-14	OLEEUCl040028:Contig2	<
N2*	278	JK784448	Heat shock protein 101 *(Glycine max);* AAA66338	1.27E-08	-	<
N3	227	JK784449	Patellin-3 protein *(Ricinus communis);* EEF40611	1.00E-24	OLEEUCl021276:Contig1	>
N6	618	JK784417	Peptide n4-(n-acetyl-β-glucosaminyl)asparagine amidase *(Vitis vinifera);* CAN73340	1.34E-59	OLEEUCl004776:Contig1	<
N7	525	JK784450	VTC2-like protein *(Actinidia chinensis);* ABP65665	5.00E-84	OLEEUCl009559:Contig1	<
N8*	403	JK784451	Purple acid phosphatase *(Solanum tuberosum);* AAT37529	7.24E-36	-	<
N9	348	JK784418	Protein S *(Catharanthus roseus);* AAU95203	3.00E-22	-	LP<
N11*	206	JK784452	Protein S *(Catharanthus roseus);* AAU95203	6.57E-05	OLEEUCl003505:Contig1	HP <
N13	157	JK784453	Pathogenesis-related protein 1 *(Vitis vinifera);* XP_002274307	1.69E-08	-	HP <
N14	500	JK811340	Pheophorbide A oxygenase *(Ricinus communis);* XP_002533389	1.48E-44	OLEEUCl057548:Contig1	<
N15	420	JK784454	Transcription elongation factor B *(Ricinus communis);* EEF41906	8.00E-16	OLEEUCl063421:Contig1	LP >
N16*	290	JK784455	Phosphoethanolamine n-methyltransferase *(Vitis vinifera);* CBI26879	6.20E-27	OLEEUCl008056:Contig1	LP <
N19	168	JK784457	Zinc finger protein *(Ricinus communis);* XP_002509838	2.95E-05	E8NTSAO03FTDF6	<
N20	555	JK784458	Ferric reductase-like transmembrane component *(Vitis vinifera);* CBI29608	1.13E-10	OLEEUCl014902:Contig1	<
N21*	494	JK784459	**Endo-1.3-β-glucosidase precursor** *(Ricinus communis);* EEF37068	1.00E-77	OLEEUCl049037:Contig1	HP <
N22	404	JK784460	Histone H3 *(Vitis vinifera);* CBI23573	1.96E-49	OLEEUCl010285:Contig1	HP <
N24	288	JK784461	RelA-SpoT like protein RSH4 *(Nicotiana tabacum);* BAC76005	3.00E-33	OLEEUCl045031:Contig1	<
N25	213	JK784462	Serine hydroxymethyltransferase *(Gossypium hirsutum);* ACJ11726	4.37E-01	OLEEUCl009950:Contig1	HP =
O1*	441	JK784419	Seed maturation protein *(Ricinus communis);* XP_002521590	5.80E-25	OLEEUCl043272:Contig1	LP>
O2	397	JK784420	Plant regulatory factor 7 *(Petroselinum crispum);* CAC00658	1.64E-03	E8NTSAO01AHUV3	LP <
O3*	348	JK784421	AP2 -erf domain-containing transcription factor *(Vitis vinifera);* XP_002283864	1.25E-03	-	HP<
O4*	327	JK784422	Xylem serine proteinase 1 *(Vitis vinifera);* CBI35805	9.47E-20	OLEEUCl004917:Contig1	<
O6*	231	JK784423	Cytochrome b5 *(Olea europaea);* CAA04702	1.92E-21	OLEEUCl064526:Contig1	LP >
O7*	215	JK784424	**Glucan endo-β-glucosidase** *(Vitis vinifera);* CBI28862	3.02E-02	E8NTSAO04IQF48	HP <

O11	70	JK784464	Flavonol synthase *(Eustoma grandiflorum);* Q9M547	0.137242	OLEEUCl011185:Contig1	<
O12*	204	JK784427	14-3-3 like protein *(Platanus x acerifolia);* CAQ16340	2.04E-06	E8NTSAO02EGALO	>
P1*	182	JK784428	Epoxide hydrolase *(Nicotiana benthamiana);* ACE82566	1.15E-12	OLEEUCl041345:Contig1	> <
P2	327	JK784429	Chlorophyll a-b binding protein 6a *(Solanum lycopersicum);* AAA34140	3.03E-34	OLEEUCl010556:Contig1	<
P5	181	JK784465	DNA binding protein *(Ricinus communis);* XP_002511487	2.03E-03	OLEEUCl034105:Contig1	LP<
P6[-]	219	JK784432	Mitochondrial carrier protein *(Arabidopsis thaliana);* AAD21477	6.00E-29	-	LP <
P7*	200	JK784433	Pathogenesis related protein *(Vitis vinifera);* CBJ49379	5.80E-01	OLEEUCl044846:Contig2	<
P11	152	JK784435	Receptor protein kinase *(Ricinus communis);* XP_002520456	2.91E-08	-	<
P13*	227	JK784436	Ferrodoxin precursor *(Capsicum annuum);* Q9ZTS2	2.29E-05	OLEEUCl007830:Contig3	<
P14*	196	JK784437	Polygalacturonase *(Solanum lycopersicum);* AAB09576	6.00E-25	OLEEUCl020995:Contig2	>
P16*	187	JK784438	**Copper amine oxidase** *(Arabidopsis thaliana);* BAD95322	1.22E-22	E8NTSAO04IL1YP	HP >

[a] Genebank accession number is provided.
[b]Homolog of best hit from BLASTX search using TDF is provided. Annotation, species and gene bank accession number are provided. In bold are indicated candidate transcripts involved in biophenol synthesis or degradation. validated by Real-Time PCR.
[c] The E-value from the best hit of the BLAST is provided. It was used to indicate the significance of sequence similarity.
[d] Identification number (ID) of the homologue (EST or cluster) in OLEA database (*http://140.164.45.140/oleaestdb/*).
[e] TDFs can be preferentially expressed in cultivar with high polyphenol content (HP). in cultivar with low polyphenol content (LP). the expression increase (>). decrease (<). increase and decrease (> <) or is constant during fruit ripening (=).
* cDNA-AFLP fragments for which the expression profile was confirmed by RT-sqPCR.
[-] cDNA-AFLP fragments for which the expression profile was not confimed by RT-sqPCR.
