# Supplementary material for: Olive phenolic compounds: metabolic and transcriptional profiling during fruit development
Source: BMC Plant Biol. 2012 Sep 10;12:162. doi: 10.1186/1471-2229-12-162 (PMC3480905; doi:10.1186/1471-2229-12-162)
Supplement: Additional file 12 — Primers used for the amplification of transcripts involved in secondary metabolite synthesis in olive. Primer sequences and amplicon size are provided. [file 1471-2229-12-162-S12.pdf]

Additional file 12 - Primers used for the amplification of transcripts involved in secondary metabolite synthesis in olive.

Transcript	Forward	Reverse	Amplicon size (bp)
OeDXS	AGACGAAGGCGAAAACCCCTAC	ATTTCCGCGGCTCCTAGACATT	650
OeDXR	GCATTCTTGGTTGAGGGATTGC	TCTGAATGCACCTCCTGAAGCA	717
OeCDPMES	AAATCTCTCTCCGGGTGGTTGA	CAGAAACATGGTGAAACTAAAATGAGG	1144
OeCDPMEK	GGGGGAGAAGCAAATTTCGAG	CAGCTGTGGAACATGCCTCTTG	904
OeMECPS	TGGCTATGGCGACTTCAGCTC	GGCTGAAGGGTCTGCACCAA	602
OeHMBPPS	TGTTGCGTTAGAAGAGTTGCCTG	CAATGCAATAGGAGGTGCGAAA	1786
OeHMBPPR	CCGGACCGGACTTCTTGTTG	GCCGTAAAACATAGACCTGCACA	1408
OeIPPI	TAGACTACAGTATTTGATTTGGC	GTCACCCATAGCGGTGGAGG	892
OeHMGR	GCCAGCACAAACAGGGGTTG	TCCTGTCAGCCTCTGCAACG	211
OeMVAK	GGTTCACGGATCCACTGCTG	TCGAGTTTCACTCCAATTACAGG	1377
OeMVAPK	CAACTGCTGTTGTTGCTGCTTT	CAAAGACGTAAGTCACTACCCAAAAA	402
OeMVAPPD	GCAGAGTACGTCGGGGGAGA	GGCCGTTATTTTCAGAATTTGATG	1071
OeGES	GCCTTATGCTTCCGCTTGCT	TGGCATTGCATTTGGTGAAA	1220
OeGE10H	TCACCCAAGGATTTGGATATGGA	AATCGTCCCCGCGTACTCTG	1468
OeSLS1	GACCTCGCATGTGCATTGGA	TGGATGAGGAGACGGCTTTTG	1024
OeSLS2	TTCCTCCTGTAATGGAATGTTTTC	TCTTTTGAATCTCCCCGCGT	1503
OeSLS3	TGGAAAGAGTTGGCTTTCAAGGA	TGATAGGGGCTCCAAACTGAGG	959
OeSLS4	TCATGCAGAAGCCTGGTCAAAA	GAACAAATGCAGTGGTGTCATGC	803
OeLAMT	*CGGTGGAGACAGCGAAAGAAAT*	*CGAGTCTACTTTGGCTGCATCG*	686
OeNDHD	AACCCATGGCAGCAACTTCAA	CATCTTGGATTTGGCCATCG	621
OeGT	GGCGACATCACCAGTTGCAT	TCGTGTTCAAACAGCAGGGTTC	1326
OeADH	TACACGGGCCGAGACTTTCTTG	CTTTGATTTGGACACCGGCAAC	437
OeCuAO	AGATACAGGCGCGTTTTGATCG	TGCTTCAATTTTCCCATCGTG	1780
OePPO	TTTCGTGGTGGATTTGGCAG	GTCTGAGCGTCATGGGCATC	1582
OeTYRD	GGGCTCGATCTTCCTGTTCAAA	ATCGTCTTCCAGGCCAAAATGA	1508
OeALDH1	TCCCATTTGCAACCAGGTATCC	GGACCCCTTGATAAACAAGCACA	1230
OeALDH2	GCAAGGATCCCGAAGGTCTCTT	CCAATTACTCGGGAAGCACCAG	506
OePAL	TGGAATGGCGAACCTCTTCC	TTGCTGAATGAGGCAGTGCC	1695
Oe4CL	CTTTCGTCGGTCCGTACGGT	TCATCGGACCTTACCCGACG	425
OeLS	GGAAGCGCAATGGAGTTACA	ATTGTTCAAGTTCTTCAAGAGAGCCA	194
OeFPPS	TTTTTGTTTTAAGAATCTTGGAATTGA	GTCCAGCATCCGTTCAACCC	1147
OeSQS	TGTGGCTGGACTTGTTGGGTTA	TTCAGCATGCGAGAGAAATCA	517
OeGGPS	TGCTCCATTGATTGCTTTGGC	AAAGGCTCTTGCAACAAAACTGC	797
OeGLU	TCGTCAGACAAACCGGGTCA	TTAAGACCCTCCGCCGTGAA	490
OePOX	TCCTCAATCCTAATGGCTTCCA	CCCAAAGAAGGCAACATTATCA	1060
