# Supplementary material for: Olive phenolic compounds: metabolic and transcriptional profiling during fruit development
Source: BMC Plant Biol. 2012 Sep 10;12:162. doi: 10.1186/1471-2229-12-162 (PMC3480905; doi:10.1186/1471-2229-12-162)
Supplement: Additional file 13 — Primers used for RT-sqPCR analyses. Primer sequences and amplicon sizes are provided. [file 1471-2229-12-162-S13.pdf]

Additional file 13 - Primers used for RT-sqPCR analyses.

TDF	Forward	Reverse	Amplicon size (bp)
L1	GAATTCATGGATGCGAG	AATTCATTGTGGGGGG	180
L3	GAATTCGCGATCCTCT	TTAAATATGCTGTATCCG	317
L5	GAATTCGCGGAGAGAG	TTAATTCAGCACCTATTAT	200
L8	GAATTCAGACCGGACA	TTAATTGCACGAGTGGT	284
L13	GAATTCGCGAGAGAGA	TTAAATATGGCATAAGTGC	226
M1	GAATTCGCCCAAAACCA	TTAAATTCACCTGTTCCC	500
M7	GAATTCGCCAAGAATATT	TTAATTTGGGTACTGTCC	317
N2	GAATTCGCCTCTGTATT	TTAAGACCTATGGCCG	278
N8	GAATTCGCCCACTTCT	TTAACTGGTGAACATGAT	403
N11	GAATTCGCCGGTATCG	TTAACTGTGGGTCCAAA	206
N16	GAATTCGTCACTAAATTG	TTAAATAAAGCAGGTTTGT	290
N21	GAATTCGTCAGACAAAC	TTAAGACCCTCCGCC	494
O1	GAATTCGTCTAAGGACAC	TTAATTACCATTTTCTTAC	441
O3	GAATTCGTCCAACGGC	TTAATTTCCCTCAACTTA	349
O4	GAATTCGTCGACCGTG	TTAATTGTCACTTCATACA	327
O6	GAATTCGTCCATCATTG	TTAATTCTTCATAAAATGAG	231
O7	GAATTCGTCAAGATTTCT	TTAATTGCAGACCCCG	215
O12	GAATTCGTCAGAGAAGG	TTAATTCAGGAGCAGATT	204
P1	GAATTCATGGATGCGAG	AATTCATTGTGGGGGG	182
P6	GAATTCATGAGCATATCT	TTAATTGAGCTTTTACCC	219
P7	GAATTCATGTATGTGAAG	TTAATTTCGACACCTGG	200
P13	GAATTCATGTTCACCAC	TTAACTACTTCCTCCAC	227
P14	GAATTCATGTGGAGAAAT	TTAACTGTCACATTTTGC	196
P16	GAATTCATGTTCTTGTCG	TTAACTCGAAAGCTAGG	187
