# Supplementary material for: Olive phenolic compounds: metabolic and transcriptional profiling during fruit development
Source: BMC Plant Biol. 2012 Sep 10;12:162. doi: 10.1186/1471-2229-12-162 (PMC3480905; doi:10.1186/1471-2229-12-162)
Supplement: Additional file 14 — Primers used for RT-qPCR analyses. Primer sequences and amplicon sizes are provided. [file 1471-2229-12-162-S14.pdf]

Additional file 14 - Primers used for RT-qPCR analyses.

Transcript	Forward	Reverse	Amplicon size (bp)
OeEF1α	ACCACTGGTGGTTTTGAAGC	GAAACCAGAGATGGGGACAA	234
OeDXS	GACGTTGCATACATGGCTTG	AGCTTGGCCTGTCGTCTATG	112
OeDXR	TCTTTGCAAGCTTGGGTCTC	CAAACGCATATCAGGCCAC	107
OeCDPMES	ACTGCAGTTCCAGCCCATTA	CGGATGAAACTCGAAATTGG	104
OeCDPMEK	CAGAATCACATGGCTTCTTCCAG	TCGAACCCCTCTTTCAAAACAA	156
OeMECPS	CGCTGATCATCGGTGGGATTA	CGCGCCTTTCCATTTAGGAT	164
OeMECPS	CAAAGAAGCTGTCCGGCTAA	CTGAAGGGTCTGCACCAAGT	146
OeHMBPPS	GCTATGGAGCATGCAACAGAT	CCAAAATCTGCATCAGCCAT	108
OeHMBPPR	TGGTATTGCAAATCAAACAACAATG	ACCAGCTTATACATGGCATCTTG	174
OeIPPI	TTTGAATTTGCTCAGCTTTTGACA	CAAGAAACTGAAAATCTGCCATGAA	122
OeHMGR	ACCACGGAAGGATGCCTTGT	ATCGAACCCCTCCAACGGTA	154
OeMVAK	CAAATTAACAGGGGCTGGTG	CATGATTCCAACTCCGCAAT	105
OeMVAPK	TGTACGGTTTTCTCCGGAAGTG	TCATTCCGATGTCCAAATCCA	150
OeMVAPPD	ACTGCTTCGACATGCTTCAGT	TCAAATCCATGAAGAAGCCTTAAA	138
OeGES	CTTTGCCCGGGACAGACC	TTCCTCCATTTTGCCATATGTGT	154
OeGE10H	CAACTTCGAGCACACTGGAA	TTCTGGATTTTCTTGGCTGG	101
OeSLS1	GTGAAGTGGATGTGTGGCCT	CAACTCAAATAAAACGCCGG	90
OeSLS2	TTGCATCTATTCAGAATGGCAG	CGAATCCCTTCATATGACCG	98
OeSLS3	CCAACGGAGAGAAATCGAAA	CGCCTTCCTTTATTGCCTTT	100
OeSLS4	TTCGCTTTGAAGGAGAGGAA	CTTGATCGCTGCATTTCAGA	111
OeLAMT	TCGTTTCACAAACGCCAGT	TCCATGCGGGAGAAGAGTTA	118
OeNDHD	GCAGCCTATATCGACCCTGA	TGCAGGTTTCATCTGTCCAC	109
OeGT	GGTTGCTTCGTGACACATTG	TCTTGATCTCGTCGCTCTCA	187
OeADH	GTTTATCACGCACACGATGG	GCCTCAAACGCTAAATCCAA	198
OeCuAO	AAGATGGCCTTGGGAAGAAT	TTCTGCCAATCCTGTTCTCC	191
OePPO	CAATCACCCGCAGCAAAGAC	GAATGGGAGAGCAAAAGTTGGG	160
OePPO	TGATGAAAATAAACAGCTTGTGCG	AGAAAGCTGGGTCTCGACCC	159
OeTYRD	TGGATCTGACCAAACCCACT	TTAACCCGAATGCAGTAGCC	110
OeALDH1	TTTAAGTGGGGAGCTCAAATACA	GATGCTTCAGATATTCCCATGC	200
OeALDH2	GGCTGCAGGAATAGTGGAAA	CTGCCTGGTAGCAAGGAATG	83
OePAL	AATGGGGAGCTTCATCCATCA	AGAAATGTGGATGACATAAGCTTCA	155
Oe4CL	TGGATTAAGAGTTGGTGCAGC	GGATTTTCCCCATCAACTTGT	127
OeLS	AGGAACAAGATTTGTGCAAACTG	ATTGTTCAAGTTCTTCAAGAGAGCCA	166
OeFPPS	ACATCATGAAACTATCCTCAATTTTTC	TTTGTCTCTTGTAAATCTTGCCCAA	170
OeSQS	ACAGTGCCTCAATGACATGGT	TGGAATAGCACAAAACCGAAA	109
OeGGPS	GCATGATGATCTCCCTTTTATGGA	TTTTGCCACCAGCAAGAAGG	153
OeLUPS	TTGCTTGTTGGGTAGAAGATCC	CTGCATTTTCAGGCCATCTT	101
OeGLU	AAATGAAGCCGGAGCAAGTA	AAAAAGGCCGAAGTTTCGTT	181
OePOX	GGATCCTTCATCGGCAACT	TCCTGTGATGCTGCTCATCT	193
